# Lactose azocalixarene drug delivery system for the treatment of multidrug-resistant pseudomonas aeruginosa infected diabetic ulcer

**DOI:** 10.1038/s41467-022-33920-7

**Published:** 2022-10-21

**Authors:** Juan-Juan Li, Yuqing Hu, Bing Hu, Wenbo Wang, Haiqi Xu, Xin-Yue Hu, Fei Ding, Hua-Bin Li, Ke-Rang Wang, Xinge Zhang, Dong-Sheng Guo

**Affiliations:** 1 College of Chemistry, Key Laboratory of Functional Polymer Materials (Ministry of Education), State Key Laboratory of Elemento-Organic Chemistry, Nankai University, 300071 Tianjin, China; 2 College of Chemistry, Key Laboratory of Functional Polymer Materials (Ministry of Education), Institute of Polymer Chemistry, Nankai University, 300071 Tianjin, China; 3 Key Laboratory of Medicinal Chemistry and Molecular Diagnosis (Ministry of Education), Key Laboratory of Chemical Biology of Hebei Province, College of chemistry and environmental science, Hebei University, 071002 Baoding, China

**Keywords:** Materials science, Supramolecular chemistry

## Abstract

Diabetic wound is one of the most intractable chronic wounds that is prone to bacterial infection. Hypoxia is an important feature in its microenvironment. However, it is challenging for antimicrobial therapy to directly apply the existing hypoxia-responsive drug delivery systems due to the active targeting deficiency and the biofilm obstacle. Herein, we customizes a hypoxia-responsive carrier, lactose-modified azocalix[4]arene (LacAC4A) with the ability to actively target and inhibit biofilm. By loading ciprofloxacin (Cip), the resultant supramolecular nanoformulation Cip@LacAC4A demonstrates enhanced antibacterial efficacy resulting from both the increased drug accumulation and the controlled release at the site of infection. When applied on diabetic wounds together with multidrug-resistant *Pseudomonas aeruginosa* infection in vivo, Cip@LacAC4A induces definitely less inflammatory infiltration than free Cip, which translates into high wound healing performance. Importantly, such design principle provides a direction for developing antimicrobial drug delivery systems.

## Abstract (web summary)

Infection is a major issue in chronic diabetic wounds. Here, the authors report on a hypoxia triggered drug delivery system to achieve higher drug levels with controlled release to treat wounds infected with multidrug-resistant pseudomonas aeruginosa and demonstrate antibacterial and anti-biofilm action

## Introduction

Diabetic wound, which is vulnerable to hyperglycemia^1^ and the microenvironment of oxidative wound^2,3^, is one of the chronic wounds that are difficult to heal. Moreover, the diabetic wound is susceptible to bacteria, then further aggravates the wound healing^4–6^. Compared with normal tissues, bacterial infections often touch off a series of changes in microenvironments including hypoxia^7,8^, lower pH^9,10^, reactive oxygen species (ROS)^11,12^, toxins^13^, enzymes^14^, temperature^15^, etc. These microenvironment characteristics have been engaged in targeted antibacterial therapy by developing the corresponding drug delivery systems (DDSs) that respond to these stimuli, primarily focused on pH and ROS^16–18^. The DDSs improved the bioavailability of antibiotics and reduced antibiotic resistance pronouncedly^19–22^. Oxygen deficiency at the bacterial infectious site leads to anaerobic glycolysis and an increase in the local acidity^23^, overexpression of the reductive enzymes (azoreductase^24–26^, nitroreductase^27–29^, and so on), increased temperature^30^, and elevated ROS level^31^. Moreover, the hypoxic characteristics of bacterial infections and the resulting strongly reductive environment constitute an important alternative target for antibacterial treatment. Therefore, it is highly in demand to develop a hypoxia-responsive DDS for antibacterial treatment as well as more diabetic wound that is difficult to heal after bacterial infections.

Currently, abundant hypoxia-responsive DDSs have been developed and widely used in antitumor treatment^32–34^, ischemic stroke^35^, cardiovascular disease^36^, diabetes^37^, and colitis^38^. However, these reported hypoxia-responsive DDSs have never been engaged in antibacterial treatment, probably due to numerous formidable obstacles in treating bacterial infections. First of all, bacterial infections are difficult to eradicate, and the remaining bacteria at the infected site will cause incurable chronic inflammation, which will further increase the difficulty of the antibacterial treatments^39,40^. Secondly, most hypoxia-responsive DDSs reported relying on the enhanced permeability and retention effect to aid the targeted delivery to tumor^41,42^. It may generate less potency through the direct transfer of these systems to the antibacterial application because such a passive target effect is generally lacking in bacterial infections^43^. Lastly and above all, a further barrier for antibacteria is the biofilm^44^. Bacterial biofilms are formed by communities that are embedded in a self-generated matrix of extracellular polymeric substances^45,46^, therefore inhibit the action of antibiotics and further increase the difficulty of treatment^47–49^. Consequently, though significant achievements about hypoxia-responsive DDSs reported, to develop one suitable for antibacteria, we still need a specific design addressing the aforementioned challenges.

**Fig. 1:**
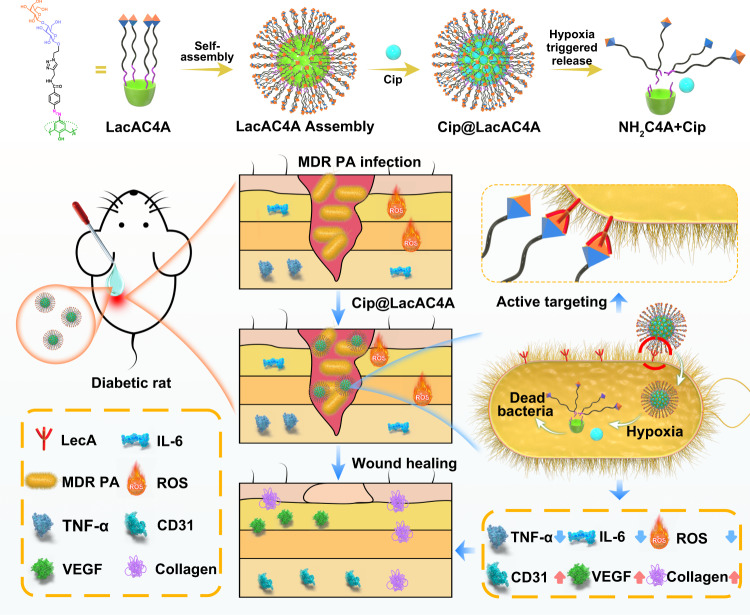
Schematic design of the hypoxia-responsive drug delivery system. The supramolecular formulation Cip@LacAC4A can actively target the MDR PA infecting diabetic rat wound via the interactions of lactose and the surface glycoproteins of bacteria. Then azo groups respond quickly under hypoxic condition, which leads to the controlled release of antibiotic for bacterial infections. It can downregulate the expression of inflammatory factors, and accelerate the wound healing.

In this work, we designed the lactose-modified azocalix[4]arene (LacAC4A) as a drug carrier customized for antibacterial purpose (Fig. 1). Lactose modification has the ability to target bacteria actively as a function of the interactions of lactose and the surface glycoproteins of bacteria. Moreover, galactose can inhibit the formation and development of biofilms by capitalizing on the observed cluster effect of binding multivalent carbohydrates to lectins. Azo groups are sensitive to azoreductase inside bacteria and therefore LacAC4A serves as a hypoxia-responsive carrier. After ciprofloxacin (Cip) was loaded in LacAC4A, the resultant gained the ability of active targeting, inhibiting biofilm and hypoxia-controlled release, which can eliminate pathogens, reduce the stimulation of immune cells, thereby downregulate the expression of inflammatory factors, and treat bacterial infections. The supramolecular formulation Cip@LacAC4A was used to treat diabetic wounds infected with multidrug-resistant *Pseudomonas aeruginosa* (MDR PA), which can realize the targeted delivery and controllable release of antibiotics, improve the antibacterial ability for drug-resistant bacteria and promote wound healing.

## Results

### Design and preparation of supramolecular nanocarriers LacAC4A and GalAC4A

**Fig. 2:**
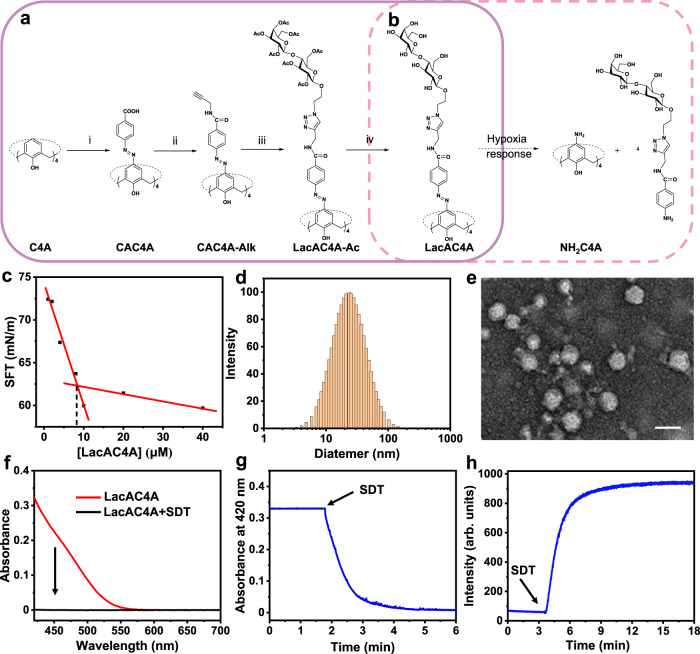
Synthesis, characterization, and hypoxia responsiveness of LacAC4A. **a** Synthetic route of LacAC4A: (i) NaNO_2_, HCl, 0–5 °C; (ii) Propargyl amine, 2-(7-azabenzotriazol-1-yl)-*N,N,N’,N’*-tetramethyluronium hexafluorophosphate, *N,N*-diisopropylethylamine, rt; (iii) Lac-N_3_, tetrahydrofuran/H_2_O, CuSO_4_·5H_2_O, L-ascorbic acid sodium salt, 55 °C; (iv) CH_3_OH, CH_3_ONa, rt; **b** The reduction reaction of LacAC4A. **c** CAC of LacAC4A. **d** DLS result of LacAC4A (0.10 mM). **e** TEM image of LacAC4A. Scale bar was 20 nm. **f** Absorbance spectra of LacAC4A (10 μM) before and after reducing by SDT (10 mM). **g** Absorbance at 420 nm of LacAC4A (10 μM) as a function of time following the addition of SDT (10 mM). **h** Fluorescence intensity at 575 nm of RhB@LacAC4A (2.0/20 μM) as a function of time following the addition of SDT (10 mM). The experiments of f-h were carried out in PBS (10 mM, pH = 7.4) at 37 °C.

LacAC4A and galactose-modified calix[4]arene (GalAC4A) were designed for the specific recognition of galactose with the galactose-specific lectin LecA on the bacterial surface and the sensitivity of their azo groups to reduce within the hypoxic bacterial microenvironment (Fig. 2a and Supplementary Fig. 6). Calixarenes were selected as the macrocyclic scaffold because of their extensive chemical design space^50–53^. By directly reacting calix[4]arene with *p*-carboxybenzenediazonium chloride at 2 °C for 3 h, CAC4A, which can be linked by condensation reaction with propargyl amine, getting CAC4A-Alk, was obtained with quantitative yield. Next, CAC4A-Alk conjugated with acetyl lactose (LacAC4A-Ac) was obtained by the click reaction of CAC4A-Alk with Lac-N_3_. Finally, the targeted compound LacAC4A was produced by the deprotection of the acetyl group. GalAC4A was obtained using the same method. (see Supplementary Information for detail).

LacAC4A and GalAC4A had similar critical aggregation concentration (CAC) values of approximately 8.0 µM as determined by the ring method of the surface tension meter (Fig. 2c and Supplementary Fig. 9). LacAC4A showed an average hydrated diameter of 25 ± 5 nm (Fig. 2d) and GalAC4A showed an average hydrated diameter of 27 ± 5 nm (Supplementary Fig. 10a), identified by dynamic light scattering (DLS) measurements. Transmission electron microscopy (TEM) of LacAC4A illustrated the spherical-like morphology with consistent size together with the DLS result (Fig. 2e). GalAC4A had similar morphology (Supplementary Fig. 10b). The amphiphilic assemblies of LacAC4A and GalAC4A provided the opportunity for multivalent interactions with LecA^54,55^.

LacAC4A solubilized Cip by grinding under the condition of a molar ratio of 1:1 (Supplementary Fig. 11). According to the phase-solubility diagram (Supplementary Fig. 12), we calculated the concentration ratio of dissolved Cip to LacAC4A as 99:100, the complexation efficiency as 99, and the binding affinity between LacAC4A and Cip was (6.70 ± 1.09) × 10^5^ M^−1^ (Supplementary Table 1). Upon loading Cip, the size distribution and morphology of Cip@LacAC4A nanoparticles remained unchanged compared with free LacAC4A, which were verified by DLS and TEM measurements (Supplementary Fig. 13a, b). Moreover, Cip@LacAC4A nanoparticles were stable for 7 days (Supplementary Fig. 13c).

To investigate the hypoxia responsiveness of LacAC4A and GalAC4A, sodium dithionite (SDT) was added into the solutions, followed by monitoring the absorbance at 420 nm of the solution continuously with a UV-Vis spectrometer. LacAC4A and GalAC4A displayed the broad absorption peaks longer than 400 nm (Supplementary Fig. 14), attributed to n-π* transitions of the azo groups according to the natural transition orbital analysis^32^. After adding excess SDT, a chemical mimic of azoreductase^56–58^, a loss of the characteristic yellow color of LacAC4A and GalAC4A was found within 10.0 min. The disappearance of the azo absorption indicated the complete reduction i.e. all four azo groups of LacAC4A were reduced (Fig. 2b, f). The reductive kinetics was quantified by monitoring the absorbance at 420 nm in real time (Fig. 2g). The attenuation curve of the intensity was well fitted in a quasi-first-order reaction decay model (Adj. R^2^ > 0.991), giving the rate constant of 1.58 min^−1^ (Supplementary Fig. 15). The half-life was calculated as 0.430 min. Therefore, the hypoxia-responsive macrocyclic amphiphilic delivery system could respond quickly under hypoxic conditions, which was a prerequisite for controllable release.

Rhodamine B (RhB**)** was employed as a model fluorescent probe to bind with LacAC4A to replace the Cip for investigating the release kinetics. The binding affinity of LacAC4A with RhB was determined to be (1.96 ± 0.12) × 10^6^ M^−1^ (Supplementary Fig. 16), which was considered to be equivalent to that of Cip@LacAC4A ((6.70 ± 1.09) × 10^5^ M^−1^). The fluorescence intensity of RhB was gradually restored in a time-dependent manner and reached saturation in 5.00 min after adding SDT to the RhB@LacAC4A complex (Fig. 2h and Supplementary Fig. 18). We evaluated the release kinetics and obtained a rate constant of 0.800 min^−1^, as well as a half-life of 0.870 min (Supplementary Fig. 19). It was reasonable to assume that the release kinetics of RhB@LacAC4A could be deduced from that of the Cip@LacAC4A system based on their comparable binding affinities. GalAC4A had a similar reduction and release kinetics (Supplementary Fig. 17 and Supplementary Fig. 21). Besides SDT, LacAC4A could also be effectively reduced by reduced nicotinamide adenine dinucleotide phosphate (NADPH) in the presence of rat liver microsomes. The rat liver microsomes are considered to represent a hypoxia-like living system, which is known to contain a wide range of redox enzymes^59^. So is it for the release of RhB from RhB@LacAC4A (Supplementary Fig. 20).

### The recognition of LacAC4A and GalAC4A towards bacteria in vitro

**Fig. 3:**
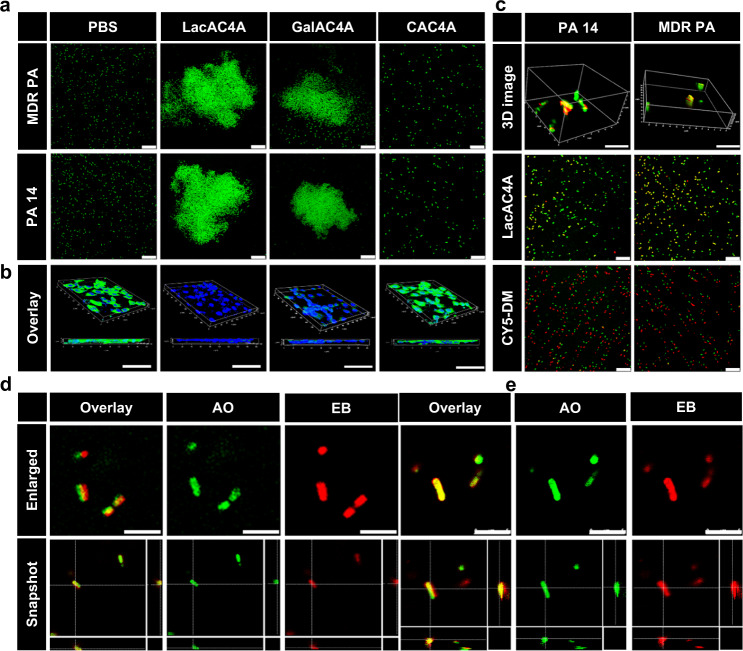
The bacterial recognition and internalization of LacAC4A. **a** CLSM images of the bacteria distribution after being treated with PBS, LacAC4A, GalAC4A, and CAC4A, respectively. Scale bar was 25 μm. **b** CLSM images of the NIH 3T3 cells infected by MDR PA after being treated by PBS, LacAC4A, GalAC4A, and CAC4A, respectively. Scale bar was 50 μm. **c** CLSM images of LacAC4A and CY5-DM internalized into bacteria. Scale bar was 10 μm in 3D images and 25 μm in other images. **d** The enlarged and snapshot images of LacAC4A internalized into MDR PA. Scale bar was 5 μm. **e** The enlarged and snapshot images of LacAC4A internalized into PA 14. Scale bar was 5 μm.

The LecA on the surface of *Pseudomonas aeruginosa* (*P. aeruginosa*) can bind with galactose specifically^60^, which was utilized to recognize *P. aeruginosa*. To evaluate the key role of galactose recognition to the LecA, *Pseudomonas aeruginosa 14* (PA 14) and MDR PA were incubated with LacAC4A, CAC4A, and GalAC4A by confocal laser scanning microscope (CLSM), respectively. As shown in Fig. 3a, bacteria in the PBS group presented a state of dispersion, while obvious bacterial clusters were observed after being treated with LacAC4A and GalAC4A. These phenomena indicated that LacAC4A and GalAC4A had a strong binding force with PA14 and MDR PA. However, some scattered bacteria could still be observed in the GalAC4A group, suggesting that the lactose groups had the stronger binding force to LecA compared with galactose groups. Meanwhile, there were no clusters occurred in the CAC4A group, showing that the binding force came from the galactose groups, not the calixarene scaffold.

### The antibacterial adhesion assay of LacAC4A and GalAC4A in vitro

The complexation of LecA in bacteria with LacAC4A hindered the bacteria from invading the host cells, thereby avoiding the production of related inflammation. Images of murine fibroblast cells (NIH 3T3 cells) infected by MDR PA were captured by CLSM after being treated with PBS, LacAC4A, GalAC4A, and CAC4A (Fig. 3b). The PBS group emerged extensive bacteria around NIH 3T3 cells, presenting bright green fluorescence, while virtually no green fluorescence was observed in the LacAC4A group, demonstrating LacAC4A could dispute the bacteria to bind NIH 3T3 cells in a competitive-binding format through the interactions between lactose groups and LecA. The GalAC4A group showed more viable bacteria than that in the LacAC4A group, while as the control of the CAC4A group, a mass of bacteria were observed, indicating the specific recognition of the glycan on the surface of nanoparticles towards bacterium cellular lectins was provided. We therefore screened out LacAC4A as the supramolecular carrier for delivering Cip due to the strong binding with Cip, the ability to capture bacteria and disrupt the bacterial adhesion to host cells.

### The internalization of LacAC4A as nanocarrier into bacteria in vitro

Typical quinolone antibiotics (Cip) can penetrate the bacterial cell membrane through the specific porin channel, and then inhibit bacterial DNA gyrase and the topoisomerase IV, thereby interfering with DNA synthesis^61^. However, the low outer membrane permeability, the intrinsic resistance of *P. aeruginosa*, is a hurdle for most antibiotics that must be overcome to treat *P. aeruginosa* infections. It prevents antibiotics from entering bacteria and exerts an antibacterial effect^18,62^.

To evaluate whether LacAC4A as a vehicle plays a positive role in the internalization of Cip into bacteria, 1,1′,3,3,3′,3′-hexamethylindodicarbocyanine (CY5-DM, a commercially available dye that could be complexed by LacAC4A with a dramatic fluorescence quenching) as a probe was applied tracing in time the transport of LacAC4A into bacteria, and PA 14 and MDR PA were incubated in vitro and observed under CLSM. The green fluorescence signal emitted by acridine orange (AO, a dye that staining the living bacteria) and the red fluorescence signal emitted by CY5-DM appeared concurrently in the bacteria after being treated with LacAC4A (Fig. 3c). This favorable overlap could be seen in the 3D images and the snapshots more clearly (Fig. 3d, e), indicating that LacAC4A could be internalized into the bacteria after specific recognition, which benefited the carrier to deliver antibiotics into the bacteria. The internalization ability of LacAC4A was further quantitatively validated by Pearson statistics, giving the Pearson correlation coefficients of more than 0.80 through calculating the ratio of overlapped green fluorescence to all green fluorescence. In comparison, free CY5-DM showed almost no overlap with the green fluorescence (Fig. 3c), indicating that free CY5-DM cannot enter bacteria.

### Hypoxia-mediated drug release in vitro

**Fig. 4:**
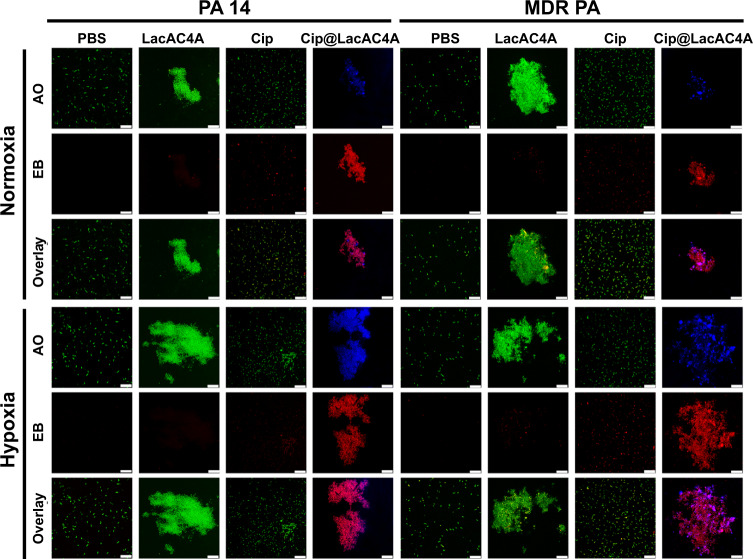
The antibacterial application and drug hypoxic release of LacAC4A. CLSM images of PA 14 and MDR PA after different treatments under normoxic and hypoxic conditions. Scale bar was 25 μm. Green represented AO, red represented EB, blue represented CY5-DM.

To observe the hypoxia response of Cip@LacAC4A in bacteria, the fluorescence images of PA 14 and MDR PA after adding PBS, LacAC4A, Cip and Cip@LacAC4A (containing 10% CY5-DM) in normoxic and hypoxic conditions were observed under CLSM in Fig. 4. When being incubated under normoxic conditions, there was no significant difference in the number of dead bacteria between Cip@LacAC4A and Cip treated PA 14, suggesting that Cip@LacAC4A and Cip had weak bactericidal activity and Cip could not be released from Cip@LacAC4A. A more number of dead PA 14 after being incubated with Cip@LacAC4A was observed via the red fluorescence of ethidium bromide (EB, a dye that staining the dead bacteria) than those being treated with PBS, LacAC4A and free Cip in hypoxic conditions, indicating that the released Cip could eliminate the bacteria. For MDR PA, the results displayed the same trend as Cip@LacAC4A emerged an excellent antibacterial activity.

The hypoxia-specific responsiveness of LacAC4A was further evaluated by incubating CY5-DM@LacAC4A with PA 14 and MDR PA. As shown in Fig. 4, after being treated with Cip@LacAC4A (containing 10% CY5-DM), the slight blue fluorescence emerged in normoxic conditions and bright blue appeared for both PA 14 and MDR PA in hypoxic conditions, indicating that CY5-DM had little release under normoxic conditions, but there was massive in hypoxic conditions in 2 h. This was because that the reductive cleavage of azo bonds was modulated by the over-expressed microbial azoreductase in hypoxia conditions^34^, followed by the release of CY5-DM displaying with the enhancement of blue fluorescence.

### The antibacterial efficiency of Cip@LacAC4A in vitro

**Fig. 5:**
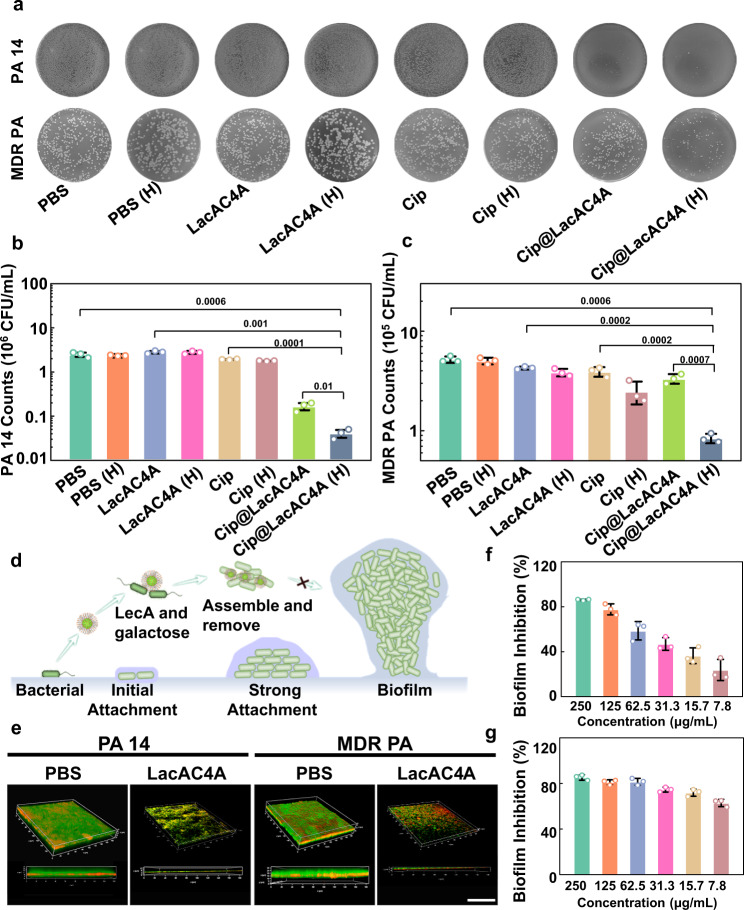
The antibacterial application and the ability to inhibit biofilm formation of LacAC4A. **a** The images of remaining bacteria after being treated with PBS, LacAC4A, Cip and Cip@LacAC4A in normoxic and hypoxic conditions, respectively. The quantification of remaining PA 14 (**b**) and MDR PA (**c**) after various treatments. **d** The mechanism schematic of LacAC4A for inhibiting the formation of bacterial biofilm. **e** CLSM images of LacAC4A for inhibiting the formations of PA 14 and MDR PA biofilms. Scale bar was 50 μm. **f** Inhibition percentage of MDR PA biofilm formation with various concentrations of LacAC4A. **g** Inhibition percentage of PA 14 biofilm formation after being treated with various concentrations of LacAC4A. Data are presented as mean ± SD, *n* = 3, *p < 0.05, **p < 0.01, ***p < 0.001.

The antibacterial nature of Cip@LacAC4A was further quantified via plate counting method. The changes in the remaining colonies after various treatments were visually displayed in Fig. 5a. Under normoxic conditions, PA 14 and MDR PA in the Cip@LacAC4A group exhibited significant reducing tendency compared with the PBS, LacAC4A and Cip groups, whereas, there were almost no bacteria occurred with Cip@LacAC4A processing in hypoxic conditions. Furthermore, the numbers of remaining colonies were quantified showing in Fig. 5b, c, the remaining bacteria treated with Cip@LacAC4A decreased in hypoxic conditions, while those remained basically unchanged in PBS, LacAC4A, and free Cip groups. This result was consistent with the Dead/Live staining, indicating that the calixarene could act as a carrier delivering Cip into bacteria, which mediated the internalization of Cip, and then improved the antibacterial ability to drug-resistant bacteria through the hypoxia-specific release of Cip from Cip@LacAC4A.

### The biofilm formation mediated by LacAC4A in vitro

Biofilm causes serious drug resistance, which is crucial to address the issue^54^. Mechanism of biofilm-mediated stress resistance includes preventing the penetration of antibiotics, changing the microenvironment to induce slow growth of biofilm cells, and inducing adaptive stress response and persistent cell differentiation^63^. As the galectin, LecA of *P. aeruginosa* can promote biofilm formation by cross-linking polysaccharide or glycoproteins and interacting with galactose-rich extracellular polysaccharides in the biofilm matrix^64^, the multivalent structure of galactose can inhibit the formation of biofilms by disturbing the activity of LecA (Fig. 5d)^65,66^. However, LacAC4A did not present the effect of dispersing the biofilm, because LacAC4A had no bactericidal ability (Supplementary Fig. 22).

To observe the inhibitory effect of LacAC4A visually on the formation of biofilms, CLSM was applied to observe the ultrastructure of biofilm after co-incubation with LacAC4A. As shown in Fig. 5e, the biofilm in the PBS group had a dense three-dimensional structure with a thickness of up to 25 μm. However, after being treated with LacAC4A, the biofilm presented a dispersed state, the number of bacteria was significantly reduced, and the thickness was obviously thinner, demonstrating the excellent inhibitory effect towards PA 14 and MDR PA.

The inhibitory ability of biofilm formation was quantified through crystal violet method in Fig. 5f, g. The inhibition of MDR PA biofilm formation by LacAC4A showed as a concentration-dependent pattern, and the inhibition rate could reach up to 80% when the concentration was 0.25 mM. A similar result was obtained in the inhibition of PA 14 biofilm formation. The inhibition rate of 80% could be achieved at a concentration of 0.25 mM.

### Evaluation of the LacAC4A biocompatibility

We employed NIH 3T3 cells to study the cytotoxicity of LacAC4A under normoxic and hypoxic conditions. As displayed in Supplementary Fig. 23, the cell viability of NIH 3T3 cells was neglectable changed and the viability stayed above 90% after being incubated with enhancing concentrations of LacAC4A for 24 h both in normoxic and hypoxic conditions, demonstrating that LacAC4A had no adverse effect on cell growth. Meanwhile, we collected major organs, including heart, liver, spleen, lung, and kidneys, and stained them with hematoxylin-eosin (H&E) for histopathologic analysis. There were no obvious tissue lesions in the main organs, reflecting the insignificant systemic toxicity of LacAC4A and Cip@LacAC4A (Supplementary Fig. 24).

### Cip@LacAC4A promoted wound healing and angiogenesis

**Fig. 6:**
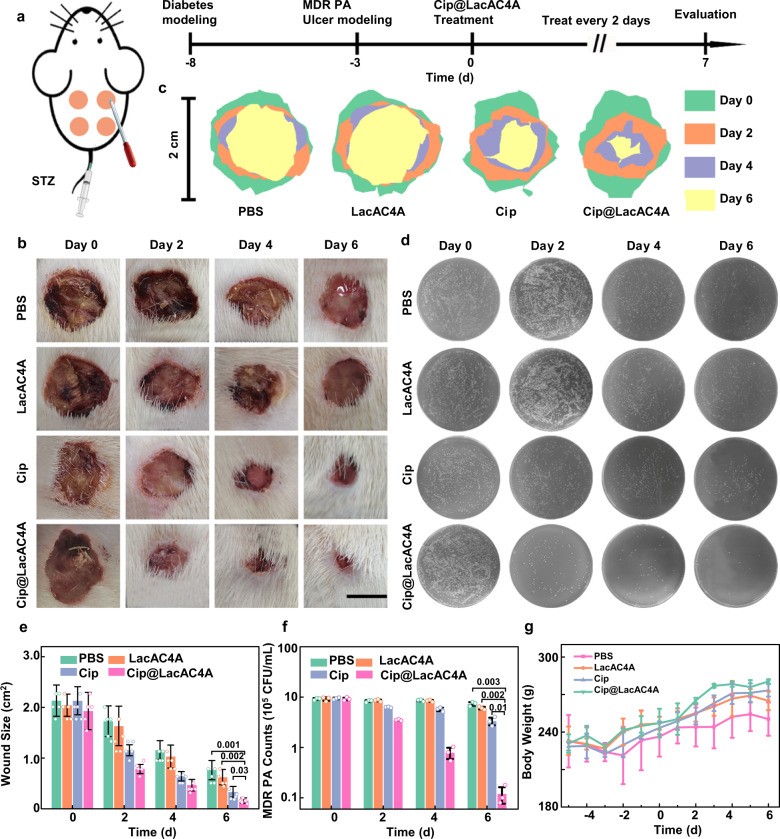
The ability of LacAC4A to promote wound healing infected by MDR PA in vivo. **a** Scheme of the therapeutic process of MDR PA-infected diabetic wound healing. **b** Representative images of wound change in diabetic rats infected with MDR PA after treatments. Scale bar was 1.0 cm. **c** Traces of wound-bed closure in diabetic rats infected with MDR PA after treatments. **d** Wound area changes in MDR PA infected diabetic rats after treatments. **e** Representative images of bacteria colonies remaining in wounds of diabetic rats infected with MDR PA after treatments. **f** Remaining bacterial changes in MDR PA infected diabetic rats after treatments. **g** Changes in body weight of rats after various treatments. Data are presented as mean ± SD, *n* = 5, *p < 0.05, **p < 0.01, ***p < 0.001.

The antibacterial capacity of Cip@LacAC4A was examined on the diabetic rat wound infected with MDR PA. The wounds associated with diabetes were more susceptible to MDR PA, which made it more difficult to control. Four 2.0 cm-diameter wounds were inflicted on the back of the diabetic rats and subsequently infected with MDR PA. Then the rats were divided into four groups randomly with various treatments: (i) PBS; (ii) LacAC4A; (iii) Cip; (iv) Cip@LacAC4A. Moreover, five healthy rats without wounds served as a negative control. The remaining bacteria and wound size were checked every two days (Fig. 6a).

The wounds shrank after the treatment of Cip and Cip@LacAC4A (Fig. 6b) and healed more rapidly in the Cip@LacAC4A group after seven days, compared with the doses in the other three groups. The changes in wound size in different groups were superimposed in situ, the rapid decrease of the wounds in the Cip@LacAC4A group provided a more intuitive explanation for the promoting effect on wound healing (Fig. 6c). The calculation of the wound area also indicated the similar results (Fig. 6d), demonstrating that Cip@LacAC4A can promote the healing of diabetic wounds, and this effect persisted in comparison with free antibiotics.

The remaining bacteria were counted via plate counting method on day 0, day 2, day 4, and day 6. The images were shown in Fig. 6e and the quantification was in Fig. 6f. As the duration of treatment time increased, the bacteria amount in PBS and LacAC4A groups remained basically unchanged with the number of bacteria at day 0, in the Cip group decreased to a certain extent (35%), but decreased significantly after being treated with Cip@LacAC4A. Almost no bacteria were detected on day 6, indicating the outstanding antibacterial nature of Cip@LacAC4A in vivo.

Additionally, the weights of rats in different groups were monitored every day shown in Fig. 6g. Weight loss on day −5 was caused by diabetes, and the corresponding decrease again after the wound modeling on day −2. As the different treatments progressed, the rats began to regain weight. Rats in the Cip@LacAC4A group returned to the initial weight on day 3, while the PBS group reached on day 6, which was consistent with the results of wound healing.

### The microenvironment changes of promoting wound healing in vivo

**Fig. 7:**
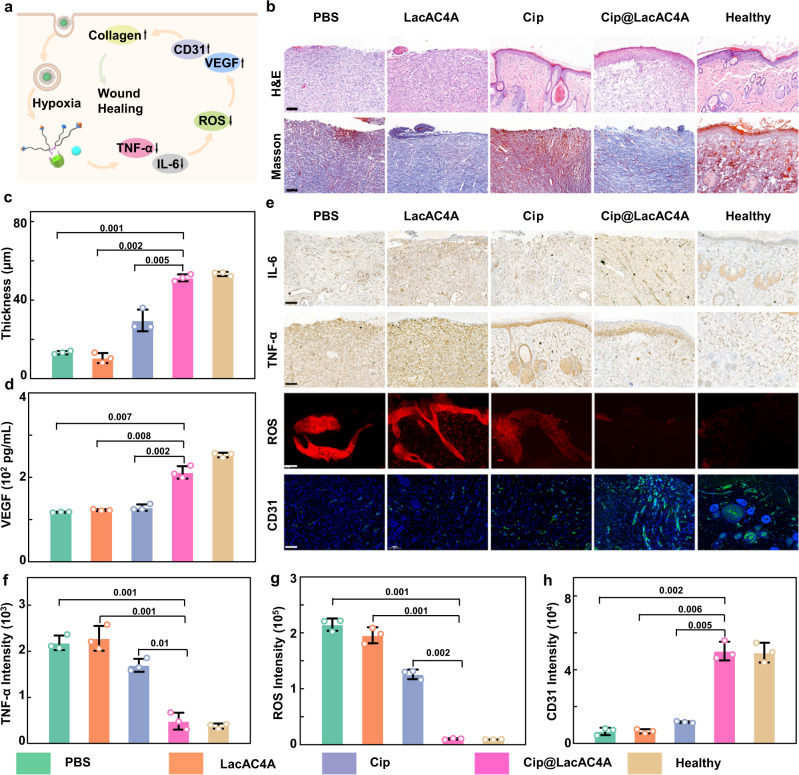
The LacAC4A promoting the diabetic ulcer wound healing. **a** The mechanism schematic of Cip@LacAC4A to promote diabetic wound healing in vivo. **b** H&E and Masson staining of wound regeneration after various treatments on day 7. **c** The thickness of granulation tissue after various treatments on day 7. **d** The expression of VEGF factor after various treatments on day 7. **e** Immunohistochemistry of TNF-α and IL-6 stained and immunofluorescence of ROS and CD31-stained images. Scale bar was 100 nm. **f** Quantitative analysis of TNF-α of wound tissues on day 7. **g** Quantitative analysis of ROS of wound tissues on day 7. **h** Quantitative analysis of CD31 of wound tissues on day 7. Data are presented as mean ± SD, *n* = 3, *p < 0.05, **p < 0.01, ***p < 0.001.

Impaired diabetic wound represents a devastating and fast-growing clinical condition associated with high morbidity, mortality, and frequent co-occurrence with bacterial infections. In this wound microenvironment, the uncontrolled accumulation of ROS considerably compromises their regenerative potential. The production of ROS requires oxygen which initiates hypoxia and further accelerates the biofilm formation^67^. Meanwhile, ROS impairs its signal mediation by mediating the covalent modification of HIF-1α, thereby impairing angiogenesis^68^. At the same time, hyperglycemia accelerates the formation of acute inflammation and reduces the release of vascular endothelial growth factor (VEGF), thereby delaying wound healing^69^. Therefore, we further revealed the wound microenvironment changes of adding Cip@LacAC4A in order to promote the wound healing by measuring the changes of related factors in the process of wound healing (Fig. 7a).

On day 7, rats were sacrificed and the thickness of the granulation tissue was carried out by H&E staining which was one of the important indexes to evaluate the process of wound healing. The results in Fig. 7b showed that there were almost no granulation tissue in the PBS and LacAC4A groups, and thin granulation tissue occurred after being treated by free Cip. Among these groups, the thickest granulation tissue appeared in Cip@LacAC4A group. Furthermore, the thickness of granulation tissue in each group was measured with Cassviewer (Fig. 7c). The thickness of the Cip@LacAC4A group was obviously thicker than the other groups and was basically the same as that of the healthy group, proving that Cip@LacAC4A promoted the formation of granulation tissue. In addition, the expression level of collagen fibers in Cip@LacAC4A group was higher than that in the other groups in Masson staining (Fig. 7b), indicating that Cip@LacAC4A could promote collagen deposition to accelerate the wound healing.

VEGF is a highly specific vascular endothelial cell growth factor that promotes vascular permeability and angiogenesis, so it is applied as a key factor to demonstrate the degree of wound healing^70^. The expression of VEGF was measured by enzyme-linked immunosorbent assay (ELISA) in Fig. 7d. The expression level of VEGF in Cip@LacAC4A group was obviously superior among those in PBS, LacAC4A and Cip groups, and was comparable to that in the healthy tissue, suggesting that Cip@LacAC4A could promote the expression of VEGF, thereby accelerating the angiogenesis and wound healing.

Tumor necrosis factor -α (TNF-α) is a small molecule protein secreted by monocytes and macrophages, and interleukin-6 (IL-6) is a lymphocyte cytokine produced by activated T cells and fibroblasts^67^. The regulation of Cip@LacAC4A on the inflammatory phase was explained by measuring the expression of these two factors (Fig. 7e). The PBS and LacAC4A groups had strong inflammation expression, showing serious infection, while the expression of inflammation in the Cip group was weakened and there was almost no expression of TNF-α and IL-6 in the Cip@LacAC4A group. Image J served to further quantify the expression of TNF-α and IL-6 in Fig. 7f and Supplementary Fig. 25. The results demonstrated that Cip@LacAC4A could regulate the inflammatory period of impaired diabetic wounds by down-regulating the expression of inflammatory factors, thereby regulating inflammatory phase.

As a typical regulatory factor in impaired diabetic wounds, the intracellular ROS levels were indicated by freeze staining^5^. As shown in Fig. 7e, the strong red fluorescence representing ROS was observed in the PBS and LacAC4A groups and weaker fluorescence was shown in the Cip group, while a dramatically diminished red fluorescence was emerged in the Cip@LacAC4A group that was the same as the healthy tissue, demonstrating the excellent antioxidative performance of Cip@LacAC4A. The quantified ROS content through Image J was shown in Fig. 7g. The ROS content in Cip@LacAC4A group was significantly different from that in PBS, LacAC4A and Cip groups, illustrating that Cip@LacAC4A scavenged the excess ROS in the organisms. We presumed that the antimicrobial capacity of Cip@LacAC4A reduced the numbers of neutrophils and macrophages recruited by bacteria, then decreased the production of ROS, alleviated oxidative stress, and promoted the proliferative phase^69,71^.

The assay of immunofluorescence of platelet endothelial cell adhesion molecule-1 (CD31), a factor that accounts for angiogenesis^69^, was applied to evaluate the effect of different treatments on angiogenesis (Fig. 7e). The green fluorescence representing CD31 rarely appeared in the PBS and LacAC4A groups, and only slightly in the Cip group, but was significantly increased in the Cip@LacAC4A group. The quantitative intensity of CD31 through Image J (Fig. 7h) showed that the expression level of CD31 in Cip@LacAC4A group was much higher than those in PBS, LacAC4A and Cip groups, and the same as that in healthy group, which indicated that Cip@LacAC4A promoted the angiogenesis.

Based on the above, Cip@LacAC4A was a potential wound therapeutic agent due to its ability to regulate inflammation and eradicate bacterial infections in vivo. The process of Cip@LacAC4A in promoting the wound healing mainly included four signs of progress: First, under the hypoxic microenvironment of diabetic wounds, the azo bonds of LacAC4A were broken to release Cip; Second, the inflammatory phase was regulated by reducing the acute immune response; Third, the generation of ROS in the organisms was scavenged to promote the proliferation phase; Finally, the reduction of the damage to growth factors and extracellular matrix (ECM) to further promote angiogenesis. Meanwhile, with the help of newly formed blood vessels, cell metabolism accelerated, more collagen fibers deposited, and thereby accelerated the remodeling of newly formed tissues and eventually promoted wound healing.

## Discussion

In summary, we designed LacAC4A as a hypoxia-responsive carrier customized for antibacterial application based on the specific lactose interactions with LecA and the sensitivity of azo bond reduction. Adding lactose to the azocalixarene carrier not only enabled the carrier to actively target bacteria, but also inhibited the formation of biofilm. By loading Cip, the supramolecular nanoformulation Cip@LacAC4A could efficiently deliver Cip into bacteria and then release rapidly under hypoxic microenvironment, providing better treatment efficacy than the antibiotic alone. Furthermore, Cip@LacAC4A promoted wound healing by eliminating ROS, reducing the acute immune response and repairing damage to growth factors and ECM by bacterial infection.

In this work, we have validated an original example of applying LacAC4A as a hypoxia-responsive carrier to treat diabetic wounds triggered by *P. aeruginosa*. Similarly, LacAC4A is also suitable for other diseases involving *P. aeruginosa* infection, such as otitis externa, burns and green nail syndrome. In addition, we can replace actively targeted groups to accommodate other inflammatory diseases of bacterial infections, expanding the scope of hypoxia-responsive DDSs’ application in the antibacterial field.

## Materials

### Chemicals

All the reagents and solvents were commercially available and used as received unless otherwise specified purification. Sodium dithionite (SDT) was purchased from J&K Chemical. 4-Aminobenzoic acid, sodium nitrite and rhodamine B (RhB) were purchased from Aladdin. Male rat liver microsomes were purchased meilunbio Tech. Co.,Ltd. Dihydronicotinamide adenine dinucleotide phosphate tetrasodium salt was purchased from Ark. 1,1′,3,3,3′,3′-Hexamethylindodicarbocyanine (CY5-DM) was obtained OKeanos Tech. Co.,Ltd. 2-(7-Azabenzotriazol-1-yl)-*N,N,N*′*,N*′-tetramethyluronium hexafluorophosphate (HATU), *N,N*-diisopropylethyl-amine (DIPEA), propargulamine, copper sulfate pentahydrate (CuSO_4_·5H_2_O), sodium ascorbate and sodium methanolate (MeONa) were purchased from TCI. 5,11,17,23-Tetrakis[(p-carboxy-phenyl)azo]−25,26,27,28-tetra-hydroxy calix[4]arene (CAC4A) was synthesized. Ciprofloxacin (Cip) was purchased from YuanYe Bio-Technology Co.,Ltd. Fetal bovine serum (FBS) and Dulbecco’s modified eagle medium (DMEM) were purchased from Thermo Fisher Scientific. Rat VEGF ELISA kit was purchased from Neobioscience Technology Company. Acridine orange (AO) and 4’,6’-diamino-2-phenylindole (DAPI) were purchased from J&K Chemical. Ethidium bromide (EB) was purchased from Saen Chemical Technology Company. Murine fibroblast cells (NIH 3T3 cells) were purchased from procell life science&technology Co.,Ltd. *Pseudomonas aeruginosa* 14 (PA 14) and multidrug-resistant *Pseudomonas aeruginosa* (MDR PA) were provided by Microbial Resource Platform, College of Life Sciences, Nankai University.

### Samples

The phosphate-buffered saline (PBS) solution of pH = 7.4 was prepared by dissolving 0.603 g of sodium phosphate monobasic dehydrate, 0.870 g disodium phosphate, 8.006 g sodium chloride and 0.201 g potassium chloride in approximate 900 mL double-distilled water. Titrate to pH = 7.4 at the lab temperature of 25 °C with NaOH and make up volume to 1000 mL with double-distilled water. The pH value of the buffer solution was then verified on a pH-meter calibrated with three standard buffer solutions. The nanoparticles were prepared by dissolving LacAC4A and GalAC4A in PBS, respectively. The self-assembled LacAC4A and GalAC4A nanoparticles were formed spontaneously benefiting from their amphiphilicity. The samples of Cip@LacAC4A were prepared by grinding.

### Apparatus

^1^H and ^13^C NMR data were recorded on a Bruker AV400 spectrometer. UV-Vis spectra were recorded in a quartz cell (light path 10 mm) on a Cary 100 UV-Vis spectrophotometer equipped with a Cary dual cell peltier accessory. Fluorescence measurements were recorded in a conventional quartz cell (light path 10 mm) on Cary Eclipse and PerkinElmer FL6500. Mass spectra were performed on Fourier transform ion cyclotron resonance mass spertrometer (Varian 7.0T FTMS, MALDI) and time-of-flight mass spectrometer (AutoflexIII LRF200-CID, MALDI). High-performance liquid chromatography (HPLC) system (Waters, Alliance2695) was employed to perform chromatographic analysis. The transmission electron microscopy (TEM) sample was examined by a TEM (HITACHI HT7700 Exalens). The sample solutions for dynamic light scattering (DLS) measurements were examined on a laser light scattering spectrometer (NanoBrook 173plus). Fluorescence microscopy images were observed by a confocal laser scanning microscope (Leica TSC SP8). Organs were homogenized with a GentleMACs dissociator (FJ200-SH).

### Reporting summary

Further information on research design is available in the Nature Research Reporting Summary linked to this article.

## Supplementary information

Supplementary Information

Reporting Summary

## Data Availability

The data supporting the findings of this study are available within the paper and its Supplementary Information, and from the corresponding author upon reasonable request. Source data are provided with this paper.

## Source data

Source Data
