# Supplementary material for: Lactose azocalixarene drug delivery system for the treatment of multidrug-resistant pseudomonas aeruginosa infected diabetic ulcer
Source: Nat Commun. 2022 Oct 21;13:6279. doi: 10.1038/s41467-022-33920-7 (PMC9586954; doi:10.1038/s41467-022-33920-7)
Supplement: Supplementary file 1 — Supplementary Information [file 41467_2022_33920_MOESM1_ESM.pdf]

Supplementary Information

For

Lactose Azocalixarene Drug Delivery System for the Treatment of Multidrug-Resistant Pseudomonas Aeruginosa Infected Diabetic Ulcer

Juan-Juan Li,‡ Yuqing Hu,‡ Bing Hu, Wenbo Wang, Haiqi Xu, Xin-Yue Hu, Fei Ding, Hua-Bin Li, Ke-Rang Wang,* Xinge Zhang* and Dong-Sheng Guo*

J.-J. Li, H. Xu, X.-Y. Hu, F. Ding, H.-B. Li, Prof. D.-S. Guo

College of Chemistry, Key Laboratory of Functional Polymer Materials (Ministry of Education), State Key Laboratory of Elemento-Organic Chemistry, State Key Laboratory of Medicinal Chemical Biology, Nankai University, Tianjin 300071, China.

E-mail: dshguo@nankai.edu.cn

Y. Hu, W. Wang, Prof. X. Zhang

Key Laboratory of Functional Polymer Materials of Ministry of Education, Institute of Polymer Chemistry, College of Chemistry, Nankai University, Tianjin 300071, China
E-mail: zhangxinge@nankai.edu.cn

B. Hu, Prof. K.-R. Wang

Key Laboratory of Medicinal Chemistry and Molecular Diagnosis (Hebei University), Ministry of Education; Key Laboratory of Chemical Biology of Hebei Province, College of chemistry and environmental science, Hebei University, Baoding 071002, China.

E-mail: kerangwang@hbu.edu.cn

1 Supplementary notes

Supplementary Note 1. Data analyses of fluorescence titrations. Fluorescence titrations of LacAC4A and GalAC4A were performed in PBS (10 mM, pH = 7.4). The complexation of LacAC4A and GalAC4A with reporter dye (Rhodamine B, RhB) was measured by direct fluorescence titrations. A mixed solution containing known amounts of LacAC4A (GalAC4A) and RhB was sequentially injected into 2.50 mL RhB solution in a quartz cuvette. The dye concentrations in mixed solution and cuvette are the same to keep dye concentration constant in the course of titrations. The fluorescence intensity was measured (λ_{ex} = 554 nm for RhB) before the first addition and after every addition until a plateau was reached. By fitting the fluorescence intensity (λ_{em} = 575 nm for RhB) according to a 1:1 host-guest binding stoichiometry, the association constant was obtained.[1] The fitting of data from direct titrations was performed in a nonlinear manner, and the fitting modules were downloaded from the website of Prof. Nau's group (http://www.jacobs-university.de/ses/wnau) under the column of "Fitting Functions".

Supplementary Note 2. Solubility measurement of ciprofloxacin (Cip). The excess solid of Cip in PBS (10 mM, pH = 7.4) was shaken at 25 °C overnight, and the insoluble solid was removed by centrifugation at 12000 rpm (12563 x g) for 5.00 min. The supernatant was lyophilized and re-dissolved by acetonitrile (containing 10% acetic acid). Each sample was filtered by a 0.22 μm membrane filter, then detected by HPLC. The dissolved Cip of each sample was calculated according to the standard curve of Cip (ranging from 1.0 μM to 0.80 mM). The HPLC equipment was used Alliance 2695 system with UV detection at 277 nm for Cip. A symmetry® C18 column (5 μm, 4.6 × 250 mm) was employed. The mobile phase comprised 0.1% v/v formic acid in water (A) and methanol (B), and the following gradient elution sequence was applied: 95% – 5% A (0.00 – 10.0 min), 5% – 95% A (10.0 – 10.1 min), and 95% A (10.1 – 17.0 min) at a flow rate of 1.0 mL/min. The chromatographic experiments were carried out at 35 °C, and the injection volume was 10.0 μL in all experiments.

Supplementary Note 3. Phase-solubility curve. The methodology was based on the

solubility variation of the guest molecule (Cip) with increasing the host molecule (LacAC4A) concentration. Briefly, Same excess qualities of Cip were mixed with different ratios of LacAC4A solid in a mortar, respectively. The mixtures were grounded for 30.0 min and collected with 5.00 mL PBS in bottles. Then, let the mixtures sit for 6 h and take the supernatant into other bottles to acquired clear solutions. The supernatant was diluted with 4-fold acetonitrile solution (containing 10% acetic acid) and each sample was filtered by a 0.22 μm membrane filter, the concentrations of Cip solubilized by LacAC4A were detected by HPLC according to the standard curve ranging from 1.0 to 0.50 mM. The binding affinity (K_a) was calculated from the slope of the linear portion of the phase-solubility diagram, according to equation: K_a = slope/(S_0 (1 − slope)),[2] where S_0 is the in intrinsic solubility of Cip. The complexation efficiency (CE), which refers to the complex/free host ratio, can be obtained from the slope of the phase-solubility diagram, according to equation: CE = [GH]/[H] = slope/(1 − slope),[3] where [GH] is the concentration of dissolved complex, [H] is the concentration of dissolved free host. CE value can be used to calculate the [G]:[H] ratio, according to equation: [G]/[H] =1/(1 + (1/CE)), where [G] is the concentration of dissolved guest, [H] is the concentration of dissolved host.[4]

Supplementary Note 4. Size, morphology and stability evaluation. The diameters of LacAC4A and Cip@LacAC4A were monitored in PBS (10 mM, pH = 7.4) by dynamic light scattering (DLS).

Transmission electron microscopy (TEM) was used for the visualization of the LacAC4A and Cip@LacAC4A morphology. The LacAC4A (Cip@LacAC4A) sample was deposited on copper grid covered with an ultrathin carbon support film and dried for 2.00 min at room temperature. The excess fluid was removed with a piece of filter, and the sample was negatively stained with 2% uranyl acetate for 30 s. Then the sample was air-dried for 8 h to capture the image.

The stability of Cip@LacAC4A was monitored in PBS (10 mM, pH = 7.4) at 4 °C for 7 days by DLS.

The data represent from three independent experiments. The reproducibility was high

level.

Supplementary Note 5. Reduction kinetics of LacAC4A. The reduction kinetics of LacAC4A was detected by UV−Vis spectroscopy. Excess sodium dithionite (SDT, 10 mM) was injected into 2.50 mL of LacAC4A solution (10 μM) to monitor the time-dependent absorbance of LacAC4A at 420 nm at 37 °C.[5] Besides SDT, the time-dependent absorbance of LacAC4A was measured in PBS (10 mM, pH = 7.4) containing rat liver microsomes (3.0 mg/mL) and reduced nicotinamide adenine dinucleotide phosphate (NADPH) (40 μM) at 37 °C. Argon gas was bubbled into the solution for 45.0 min to create the hypoxic environment.

Supplementary Note 6. Release kinetics of RhB@LacAC4A. The release kinetics of LacAC4A was detected by fluorescence spectrometry. Excess SDT (10 mM) was injected into 2.50 mL of RhB@LacAC4A (2.0/20 μM) solution in a quartz cuvette to monitor the time-dependent intensity of RhB at 575 nm at 37 °C.[6] Besides SDT, the time-dependent fluorescence intensity of RhB@LacAC4A was measured in PBS (10 mM, pH = 7.4) containing rat liver microsomes (3.0 mg/mL) and NADPH (40 μM) at 37 °C. Argon gas was bubbled into the solution for 50.0 min to create the hypoxic environment.

Supplementary Note 7. MTT Assay. The cytotoxicity of LacAC4A against NIH 3T3 cells was investigated by 3-(4,5-dimethylthiazol-2-yl)-2,5-diphenyltetrazolium bromide (MTT) assay. Briefly, cells were seeded at a density of 5×10^3 cells per well (200 μL) into a 96-well plate. After adherence, the cells were treated with LacAC4A of different concentrations ranging from 0 to 0.40 mM. After incubation for 24 h under normoxic and hypoxic conditions for 24 h, the culture medium was replaced with fresh medium. Then, MTT solution was added to cells at the final concentration of 0.50 mg/mL and incubated with cells at 37 °C for 4 h. Thereafter, the medium was removed and 100 μL of DMSO was added, followed by gently shaking for 10.0 min. The absorbance was measured at 490 nm using a microplate reader (Bio-Rad, iMark, USA). The relative cell viabilities were calculated as: cell viability = (OD_{490} (samples)/OD_{490}

(control)) × 100%, where OD_{490} (control) and OD_{490} (samples) were obtained in the absence and presence of LacAC4A, respectively.

Supplementary Note 8. The recognition of LacAC4A and GalAC4A towards bacteria *in vitro*. *Pseudomonas aeruginosa 14* **(**PA 14) and multidrug-resistant *Pseudomonas aeruginosa* (MDR PA) were selected as bacteria models to evaluate the ability of LacAC4A and GalAC4A to target the bacteria. Bacteria in logarithmic growth phase were diluted with PBS to OD_{600} = 2.0. Diluted bacteria (500 μL) was added with LacAC4A (0.50 mM, 500 μL) or GalAC4A (0.50 mM, 500 μL), respectively, and then incubated at 37 °C for 2 h. After that, the bacteria were stained with acridine orange (AO) (10 μL, 1.0 mg/mL) for 20.0 min in dark, and finally dropped onto a glass slide for further observing. Fluorescent images were captured by a confocal laser scanning microscope (CLSM). The data represent from three independent experiments. The reproducibility was high level.

Supplementary Note 9. The internalization of LacAC4A as nanocarrier into bacteria *in vitro*. To ensure the interactions between bacteria and LacAC4A, PA 14 (OD_{600} = 2.0, 500 μL) and MDR PA (OD_{600} = 2.0, 500 μL) were added with CY5-DM@LacAC4A (0.50/0.50 mM, 500 μL) and CY5-DM (0.50 mM, 500 μL), respectively, and then incubated for 2 h. Then the mixtures were stained with AO (10.0 μL, 1.0 mg/mL) for 20.0 min at dark. The mixture was re-dispersed in glycerin and dropped on a glass slide. The images were obtained through CLSM. The data represent from three independent experiments. The reproducibility was high level.

Supplementary Note 10. The antibacteria adhesion assay of LacAC4A and GalAC4A *in vitro*. NIH 3T3 cells were set as the model to measure the ability of LacAC4A and GalAC4A to inhibit bacteria adhering to the host cells. Bacteria stained with 4 mg FITC was incubated overnight and diluted with PBS to OD_{600} = 1.5. NIH 3T3 cells (1×10^4 per well, 380 μL) were cultured in dulbecco's modified eagle medium overnight, then 80 μL FITC-stained MDR PA were added and incubated for 15.0 min. Then, GalAC4A (0.50 mM, 300 μL), LacAC4A (0.50 mM, 300 μL), CAC4A (0.50 mM,

300 μL) and PBS (300 μL) were added respectively and incubated for 3 h. After that, the cells were fixed with 4% paraformaldehyde solution and stained with DAPI (10.0 μL, 1.0 mg/mL) for 30.0 min in the dark. The sample was dispersed in glycerin, followed by observation using CLSM. 1% Triton X-100 was selected to lyse the cells and released the remaining bacteria, which was diluted to 10^3 with PBS and counted through the plate counting method. The data represent from three independent experiments. The statistical tests were two-sided.

Supplementary Note 11. Hypoxia-mediated drug release *in vitro*. To observe the antibacterial effect of Cip@LacAC4A intuitively, the Live/Dead staining was evaluated through CLSM. PA 14 (OD_{600} = 2.0, 500 μL) and MDR PA (OD_{600} = 2.0, 500 μL) were added with LacAC4A (0.50 mM, 500 μL), Cip (0.50 mM, 500 μL), Cip@LacAC4A (0.50/0.50 mM, 500 μL) in normoxic and hypoxic conditions, respectively, and then incubated for 4 h. Then the bacteria were stained with AO (10.0 μL, 1.0 mg/mL) and ethidium bromide (EB) (10.0 μL, 1.0 mg/mL) for 20.0 min, and dropped on a glass slide to capture images by CLSM. For plate counting method, the bacteria were diluted to 10^3 after different treatments, and evenly applied to the Luria-Bertani (LB) agar plates. The colony-forming units (CFUs) were counted until the bacteria were seen. PBS treated bacteria group was served as control. The data represent from three independent experiments. The reproducibility was high level.

Supplementary Note 12. The biofilm formation mediated by LacAC4A *in vitro*. PA 14 and MDR PA were selected as models to evaluate the ability of LacAC4A to inhibit the growth of biofilm. Bacteria in rapidly growing phase were dispersed with LB medium to OD_{600} = 0.05, and added 100 μL to each well in a 96-well plate. Then different concentrations of LacAC4A (100 μL) were added to corresponding well and incubated at 37 °C for 24 h. The suspensions were removed and 200 μL anhydrous methanol was added to fix the formed biofilm. 200 μL crystal violet was used to stain the formed biofilm and then 200 μL acetic acid solution was added to dissolve the crystal violet. The absorbance at 590 nm was measured using a UV−Vis

spectrophotometer to represent the biofilm. Meanwhile, after being fixed, the biofilm was stained with EB (1.00 mL, 1.0 mg/mL) and fluorescein isothiocyanate-coupled concanavalin A (1.00 mL, 1.0 mg/mL) at 4 °C for 15.0 min, then observed under CLSM for proving the inhibition effect of LacAC4A intuitively.

Supplementary Note 13. The biofilm dispersion assay of LacAC4A *in vitro*. PA 14 and MDR PA were selected as models to evaluate the ability of LacAC4A to disperse biofilm. Biofilm dispersion assay was carried out in 96-well plates. The preparation of PA 14 and MDR PA was by means of transferring frozen bacteria strain into a LB medium with shaking at 37 °C for 12 h. The original bacteria fluid was diluted into OD_{600}=0.02, by adding the culture medium as the seeding solution. Subsequently, seeding solution (150 μL) was injected in 96-well plates and cultured at 37 °C for 48 h. When the biofilm took shape, PBS (200 μL), LacAC4A (0.50 mM μM, 200 μL), Cip (0.50 mM, 200 μL), Cip@LacAC4A (0.50/0.50 mM, 200 μL) were added into the 96-well plates and incubated for 2 h. The biofilms were rinsed with PBS (150 μL) for three times and anhydrous methanol (200 μL) was added to each well of microtiter plate to immobilize biofilms for 30.0 min. After being rinsed with PBS (200 μL) three times to remove residual methanol, every well of 96-well plates was added crystal violet (200 μL) for 20.0 min. After the biofilms were stained by crystal violet, the 96-well plates were washed with PBS (200 μL) three times to remove unbound dye and then acetic acid (33%, 200 μL) was added. The absorbance at 590 nm was measured by microplate reader to represent the biofilm.

Supplementary Note 14. Cip@LacAC4A promoted wound healing and angiogenesis. The antibacterial property of Cip@LacAC4A *in vivo* was evaluated on a rat model of diabetic wound infected by MDR PA. Male SD rats (6 – 8 weeks old, 200 – 250 g) were applied to establish this animal model. First, the diabetic rats were modeled through the injection of streptozotocin (STZ, 50 mg/kg), the model could be determined to be successful when the blood glucose level was greater than 16.7 mmol/L for two consecutive days. Then, a round full-thickness cutaneous wound (20 mm × 20

mm) area was created on the back and 50 μL of MDR PA solution (10^8 CFU mL^{-1}) was introduced onto the wound to create the skin ulcer model. The rats were divided into four groups, including PBS, LacAC4A, Cip, Cip@LacAC4A. Moreover, five healthy rats without wounds served as negative control. PBS (10 mM, 150 μL), LacAC4A (0.50 mM, 150 μL), Cip (0.50 mM, 150 μL) and Cip@LacAC4A (0.50/0.50 mM, 150 μL) were added to the corresponding wounds once every two days. Meanwhile, the body weights of the rats were measured every day. The scabs of the wounds were torn off every two days, then ground and diluted to 10^3 with PBS and smeared on the agar plate evenly, and the degree of infection was monitored by the number of remaining colonies.

Supplementary Note 15. Immunohistological analysis. For histological analysis, the wounds were harvested after treatments, fixed in 4% formaldehyde, and embedded in paraffin. The samples were cross sectioned to 5 μm thick slices and the slices were mounted on slides for Haematoxylin-Eosin (H&E) and Masson's staining. The intracellular ROS levels were tested by staining the slices with DHE (5 μM) and washed with PBS for three times.

For immunohistochemistry (IHC) analysis, the antibodies of TNF-α and IL-6 were selected to stain tissue sections at 4 °C to text the inflammation on the wounds after different treatments. For immunofluorescence (IF) detection, the tissue was labeled with the endothelial cell adhesion molecule-1 (CD31) antibody, and the cell nucleus were stained with DAPI. Meanwhile, the ELISA kit was chosen to analyze the expression of growth factor VEGF in the grinding fluid of the corresponding tissue. The concentrations of antibodies were diluted 100 times for experiments. The data represent from three independent experiments. The reproducibility was high level.

Supplementary Note 16. Statistical analysis. Each experiment was set to three or five groups in parallel, and all data were expressed as mean ± standard deviation (SD). One-way analysis of variance (ANOVA) and Tukey's post-test were used to analyze the significant differences in the data for each group. The statistical tests were two-sided. Statistical significance was denoted as follows: * $p < 0.05$, ** $p < 0.01$, and *** $p <$

0.001.

2 Supplementary Syntheses

2.1 Synthesis of CAC4A.

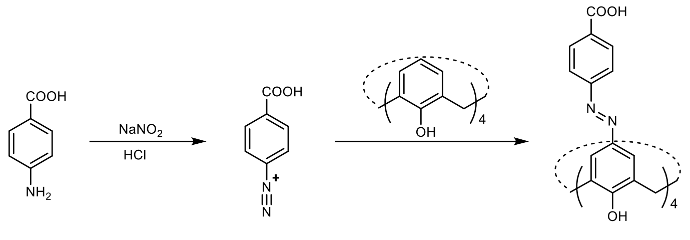

Supplementary Figure S1. The synthetic route of CAC4A.[1,7]

4-aminobenzoic acid (1.37 g, 10.0 mmol) and concentrated hydrochloric acid (2.00 mL, 37%) were dissolved in 15.0 mL water in a round bottom flask. After cooling the solution to 2 °C under an ice-water bath, a solution of sodium nitrite (10.0 mL, 10.0 mmol, in water) was slowly added, while the temperature was controlled to lower than 5 °C. The obtained solution was slowly added into a solution of 25,26,27,28-tetrahydroxycalix[4]arene (C4A, 1.00 g, 2.36 mmol) and sodium acetate (2.46 g, 30.0 mmol) in MeOH-DMF (26.0 mL, 5:8, v:v) to obtain a red suspension. After stirring at room temperature for 2 h, hydrochloric acid solution (37%) was added until the solution was adjusted to pH = 1. After warming to 60 °C for 30.0 min, the mixture was filtered and washed with water and MeOH to obtain a reddish solid of CAC4A in a quantitative yield.

^{1}H NMR (400 MHz, DMSO-d_6, δ) 8.09 (d, 8H, J = 8.0 Hz, Ar-H), 7.88 (d, 8H, J = 8.6 Hz, Ar-H), 7.88 (s, 8H, calix-Ar-H), 4.49 (s, 4H, Ar-CH2-Ar) ppm. The other bridged hydrogen is obscured by the water peak.

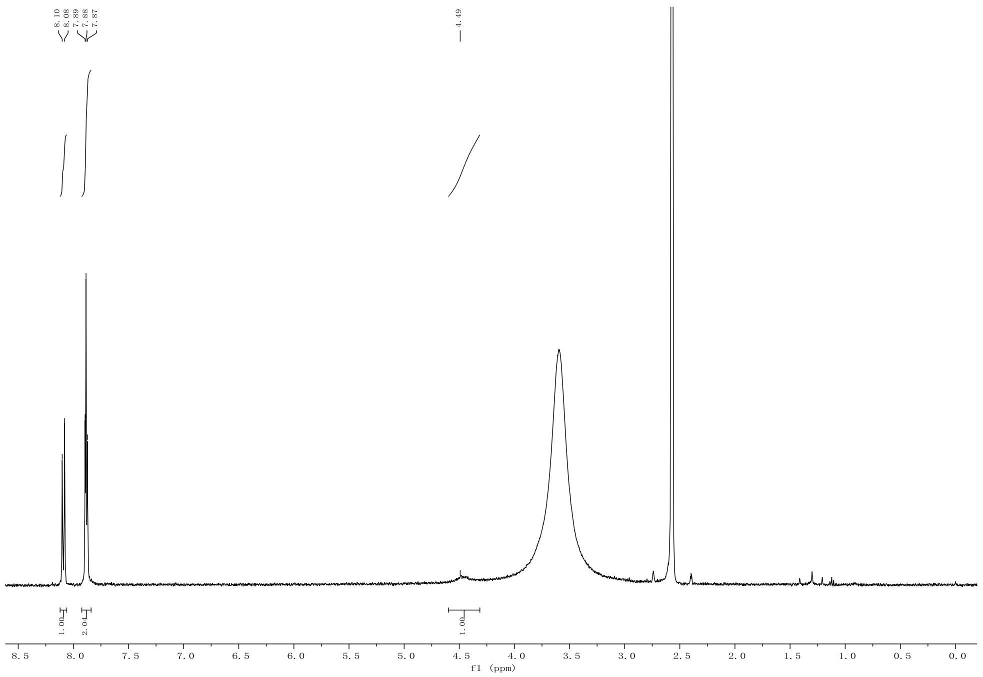

Supplementary Figure S2. ^{1}H NMR spectrum of CAC4A in DMSO-d_6, 400 MHz, 25 °C.

2.2 Synthesis of LacAC4A.

CAC4A-Alk: In a round bottom flask, CAC4A (250 mg, 0.250 mmol) and HATU (750 mg, 2.00 mmol) were added to 30.0 mL of DMF, and the reaction was placed in an ice-water bath and stirred for 30.0 min. Propargylamine (0.100 mL, 1.60 mmol) and DIPEA (0.500 mL, 3.00 mmol) were added to the reaction, react for 15.0 min under ice bath condition, and react for 6 hours at room temperature. After the reaction was completed, a large amount of water was added until a large amount of precipitate was precipitated. Then the solid was obtained by suction filtration, and the purified product was obtained after drying. CAC4A-Alk (300 mg) was obtained with a yield of 95.0%.

^{1}H NMR (400 MHz, DMSO-d_6, δ) 9.03 (t, J = 4.7 Hz, 4H, Ar-OH), 7.96 (d, 8H, J = 8.4 Hz, Ar-H), 7.81 (d, 8H, J = 9.6 Hz, Ar-H), 7.80 (s, 8H, calix-Ar-H), 4.42 (d, 4H, J = 12.0 Hz, calix-CH_2-H), 4.05-4.04 (m, 8H, Ar-CH_2-H), 3.68 (d, 4H, J = 12.0 Hz, calix-CH_2-H), 3.11 (t, J = 2.4 Hz, 4H, Ar-CH$_2$-C≡CH) ppm.

^{13}C NMR (100 MHz, DMSO-d_6, δ) 165.78, 160.27, 154.37, 144.98, 134.86, 131.17, 128.88, 124.55, 122.30, 81.77, 73.39, 54.07, 42.36, 32.39, 29.04, 18.55, 17.19, 12.96

ppm.

FTMS (MALDI): $[M+Na]^+$: *m/z* calcd. for $C_{68}H_{52}N_{12}O_8Na^+$: 1187.40, found: 1187.3960.

a

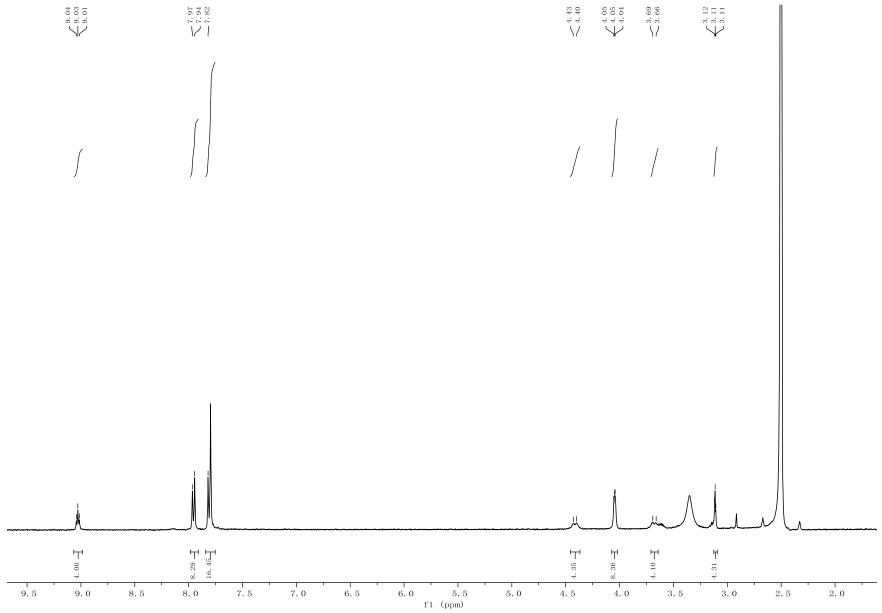

b

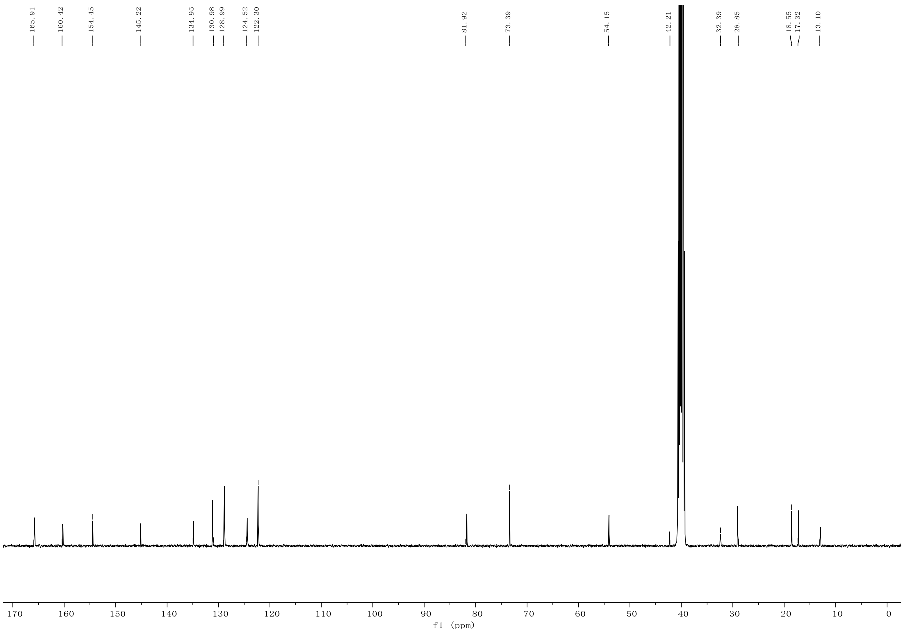

c

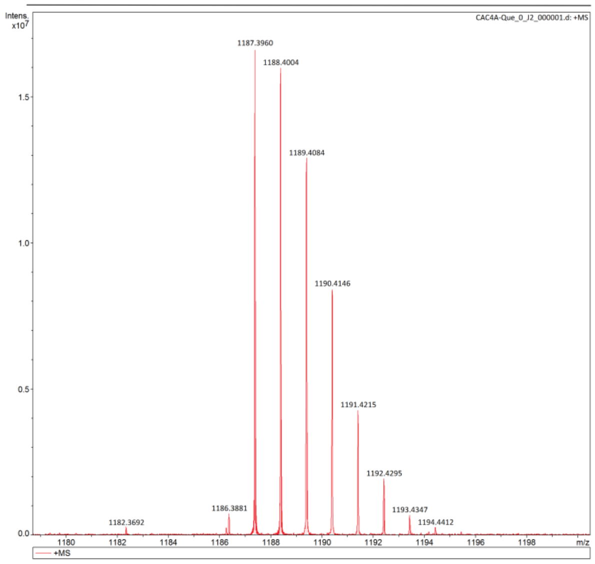

Supplementary Figure S3. Characterization of CAC4A-Alk. (a) ^{1}H NMR spectrum of CAC4A-Alk in DMSO-d_6, 400 MHz, 25 °C. (b) ^{13}C NMR spectrum of CAC4A-Alk in DMSO-d_6, 100 MHz, 25 °C. (c) FTMS of CAC4A-Alk.

LacAC4A-Ac: Add CAC4A (200 mg, 0.160 mmol) and Lac-N_3 (540 mg, 0.770 mmol) to 30.0 mL of THF in a round bottom flask. Then, a water solution of $CuSO_4 \cdot 5H_2O$ (94.0 mg, 0.380 mmol) and sodium ascorbate (76.0 mg, 0.380 mmol) was added. The reaction mixture was stirred for 10 h at 55 °C under N_2 atmosphere. The reaction solvents were evaporated under vacuum. The residue was resolved by CH_2Cl_2 and purified by silica-gel column chromatography using CH_2Cl_2/CH_3OH (v/v, 30/1) as the eluent. LacAC4A (400 mg) was obtained with a yield of 65.0%.

^{1}H NMR (400 MHz, DMSO-d_6, δ): 9.15-9.12 (m, 4H, Triazole-N*H*), 7.98 (d, 8H, J = 8.0 Hz, Ar-H), 7.87 (s, 4H, Triazole-H), 7.81 (d, 8H, J = 8.0 Hz, Ar-H), 7.80 (s, 8H, calix-Ar-H), 5.22 (d, 4H, J = 3.6 Hz), 5.18-5.14 (m, 4H), 5.10 (t, 4H, J = 9.2 Hz), 4.86-4.81 (m, 4H), 4.78-4.72 (m, 8H), 4.65-4.61 (m, 4H), 4.50-4.49 (m, 16H), 4.31 (d, 4H, J = 11.2 Hz), 4.21 (t, 4H, J = 6.8 Hz), 4.07-3.98 (m, 16H), 3.90-3.73 (m, 16H), 3.70-3.64 (m, 4H), 2.09 (s, 12H, Ac-H), 2.07 (s, 12H, Ac-H), 2.00 (s, 12H, Ac-H), 1.99 (s, 12H, Ac-H), 1.95 (s, 12H, Ac-H), 1.90 (s, 12H, Ac-H), 1.87 (s, 12H, Ac-H) ppm.

^{13}C NMR (100 MHz, DMSO-d_6, δ): 170.78, 170.37, 170.34, 169.99, 169.76, 169.55, 165.93, 160.16, 154.31, 145.11, 135.23, 131.12, 128.86, 124.43, 122.22, 100.41, 99.37, 76.70, 72.73, 72.21, 71.31, 70.75, 70.10, 69.33, 67.82, 67.48, 62.59, 61.23, 49.62, 21.07,

20.90, 20.86, 20.82, 20.77 ppm.

FTMS (MALDI): $[M+Na]^+$: m/z calcd. for $C_{180}H_{208}N_{24}O_{80}Na^+$: 4008.2900, found: 4008.2742.

a

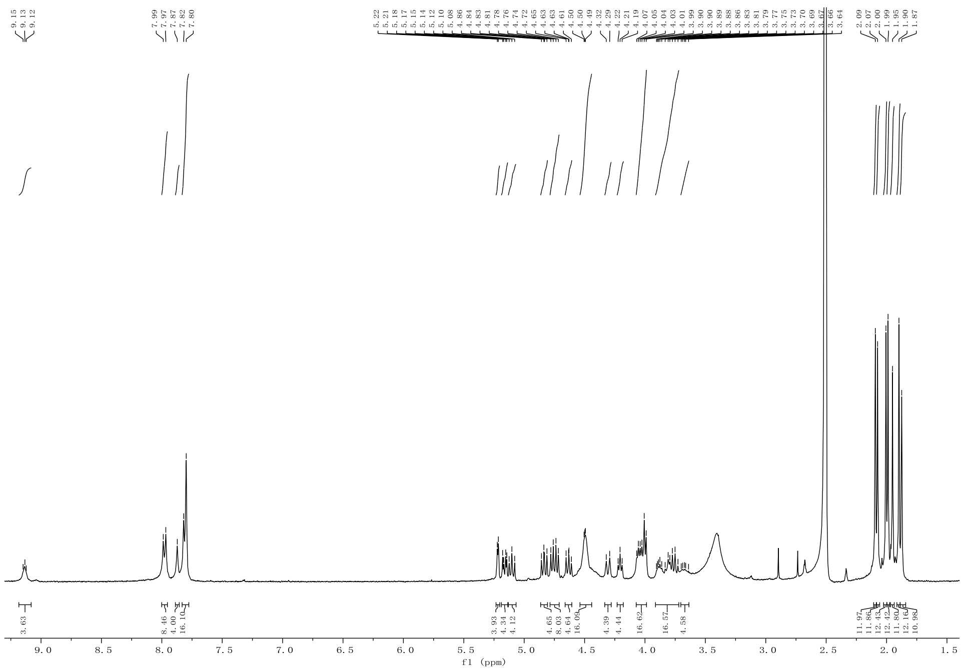

b

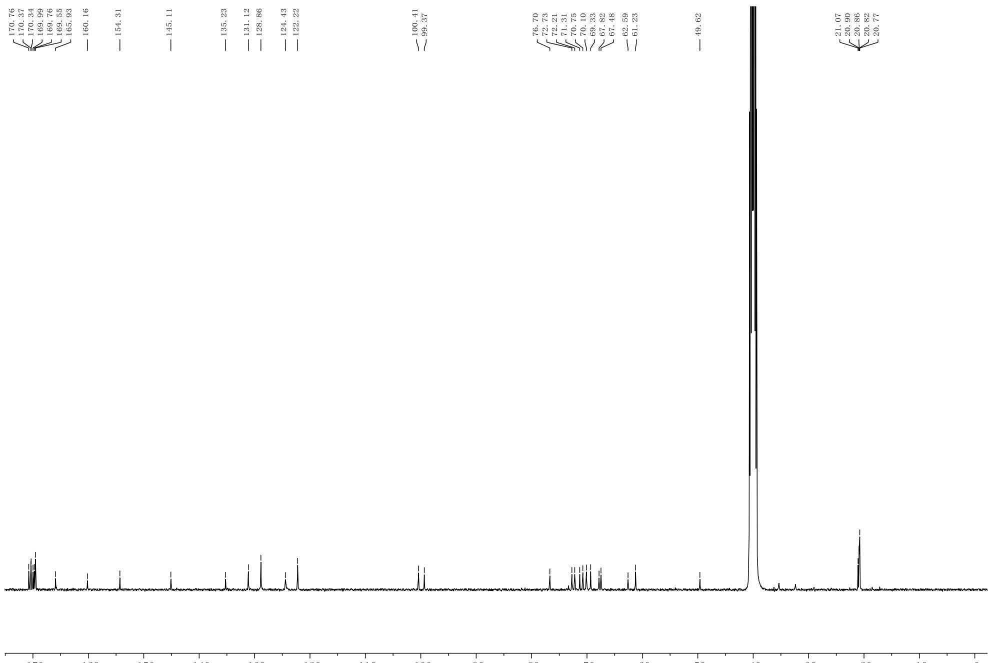

c

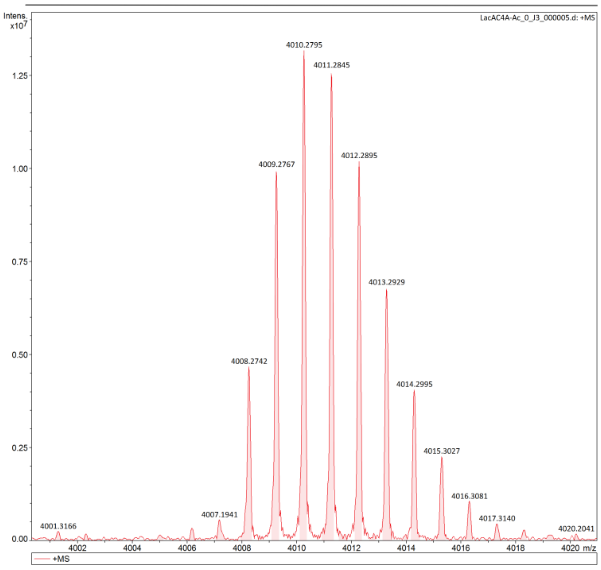

Supplementary Figure S4. Characterization of LacAC4A-Ac. (a) ^{1}H NMR spectrum of LacAC4A-Ac in DMSO-d_6, 400 MHz, 25 °C. (b) ^{13}C NMR spectrum of LacAC4A-Ac in DMSO-d_6, 100 MHz, 25 °C. (c) FTMS of LacAC4A-Ac.

LacAC4A: LacAC4A-Ac (200 mg, 0.0500 mmol) and MeONa (92.0 mg, 1.70 mmol) were dissolved in anhydrous MeOH (20.0 mL). The reaction mixture was stirred at room temperature for 10 h. The reaction mixture was placed in a cellulose dialysis tube (cutoff 2000) and dialyzed against water for 2 days. The compound LacAC4A (130 mg) was obtained through lyophilization with a yield of 90%.

^{1}H NMR (400 MHz, DMSO-d_6, δ): 9.12 (t, J = 5.6 Hz, 4H, Triazole-N*H*), 8.04 (s, 4H, Triazole-H), 7.98 (d, 8H, J = 8.0 Hz, Ar-H), 7.81 (d, 8H, J = 8.0 Hz, Ar-H), 7.80 (s, 8H, calix-Ar-H), 6.63 (s, 17H, fumaric acid-C*H*=C*H*), 5.22 (d, 4H, J = 4.8 Hz), 5.11-5.10 (m, 4H), 4.70-4.67 (m, 8H), 4.62-4.50 (m, 28H), 4.3 (d, 4H, J = 7.6 Hz), 4.18 (d, 4H, J = 6.8 Hz), 4.11-4.05 (m, 8H), 3.94-3.89 (m, 8H), 3.78-3.74 (m, 8H), 3.61-3.60 (m, 8H) ppm. The purity was 96%.

^{13}C NMR (100 MHz, DMSO-d_6, δ): 166.07, 160.32, 154.39, 145.23, 135.23, 131.28, 124.23, 122.23, 104.30, 102.92, 81.26, 75.96, 75.40, 75.32, 73.66, 73.46, 70.98, 68.60, 67.88, 60.87, 50.02, 35.36.

FTMS (MALDI): $[M+Na]^+$: *m/z* calcd. for $C_{124}H_{152}N_{24}O_{52}Na^+$: 2832.0000, found: 2831.9954.

a

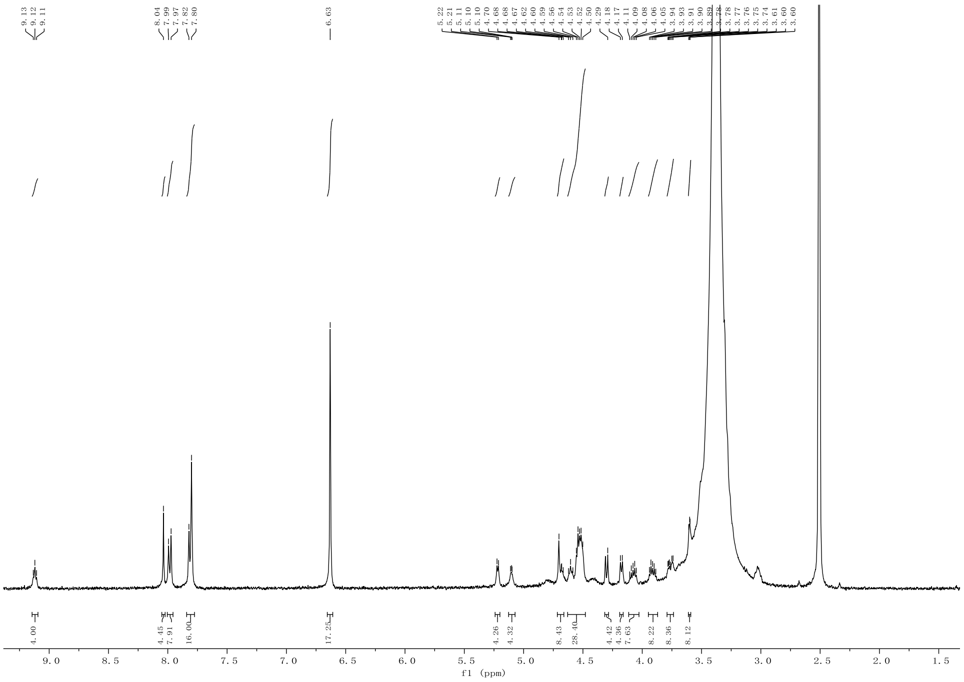
9.13
9.12
9.11
8.04
7.99
7.97
7.82
7.80
6.63
5.22
5.21
5.11
5.10
5.10
4.70
4.68
4.68
4.67
4.62
4.60
4.59
4.56
4.54
4.53
4.52
4.50
4.29
4.18
4.17
4.11
4.09
4.08
4.06
4.05
3.94
3.93
3.91
3.90
3.78
3.77
3.76
3.75
3.74
3.61
3.60
3.60
4.00
4.45
7.91
16.00
17.25
4.26
4.32
8.43
28.40
4.42
4.36
7.63
8.22
8.36
8.12
9.0
8.5
8.0
7.5
7.0
6.5
6.0
5.5
5.0
4.5
4.0
3.5
3.0
2.5
2.0
1.5
f1 (ppm)

b

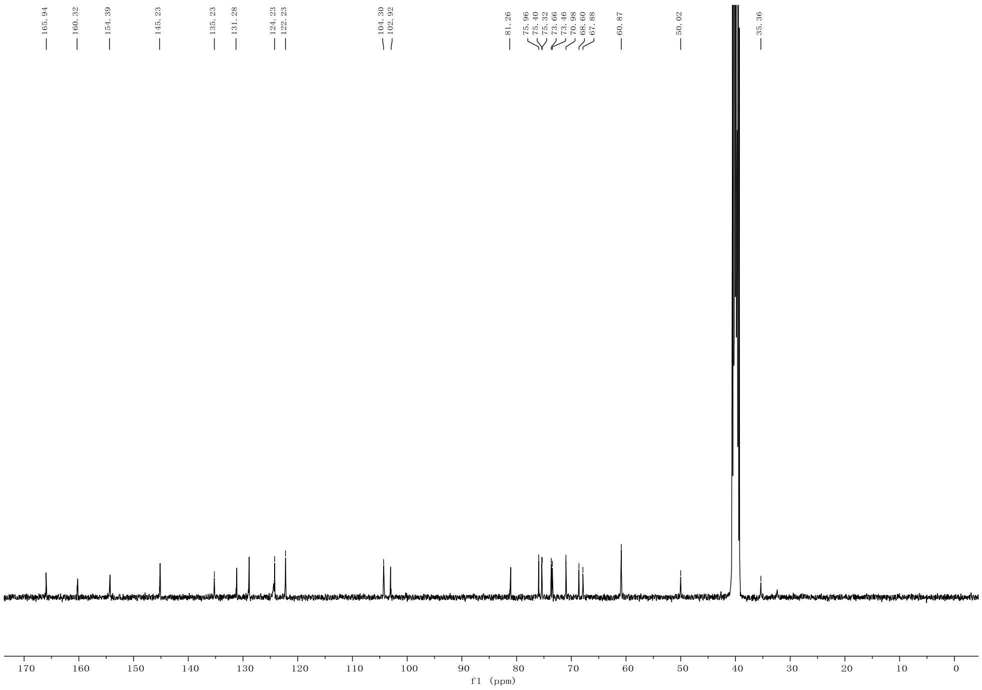
165.94
160.32
154.39
145.23
135.23
131.28
124.23
122.23
104.30
102.92
81.26
75.96
75.40
75.32
73.66
73.46
70.98
68.60
67.88
60.87
50.02
35.36
170
160
150
140
130
120
110
100
90
80
70
60
50
40
30
20
10
0
f1 (ppm)

c

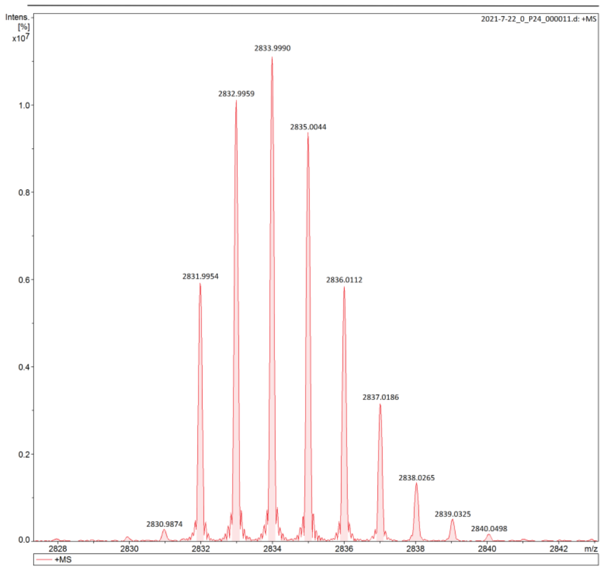

Supplementary Figure S5. Characterization of LacAC4A. (A) ^{1}H NMR spectrum of LacAC4A in DMSO-d_6, 400 MHz, 25 °C. (B) ^{13}C NMR spectrum of LacAC4A in DMSO-d_6, 100 MHz, 25 °C. (C) FTMS of LacAC4A.

2.3 Synthesis of GalAC4A.

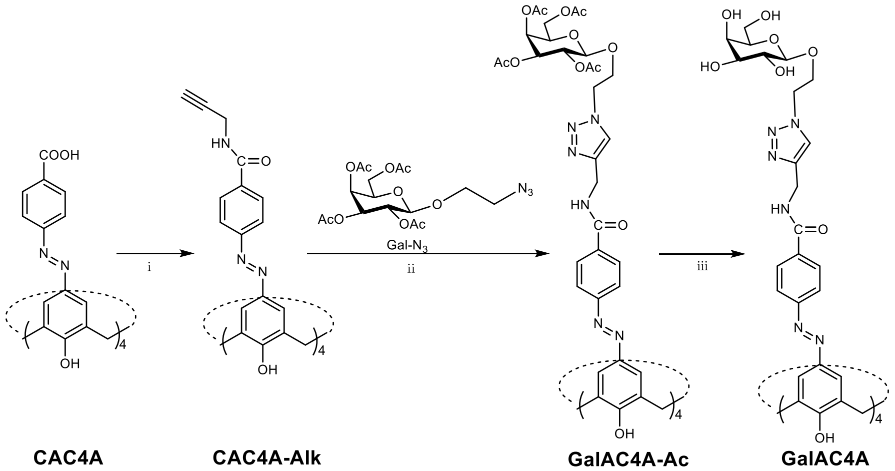

Supplementary Figure S6. (i) Propargyl amine, HATU, DIPEA, DMF, rt; (ii) Gal-N_3, THF/H_2O, $CuSO_4·5H_2O$, L-ascorbic acid sodium salt, 55°C; (iii) CH_3OH, CH_3ONa, rt;

GalAC4A-Ac: Add CAC4A-Alk (200 mg, 0.160 mmol) and Gal-N_3 (320 mg, 0.770 mmol) to 30.0 mL of THF in a round bottom flask. Then, a water solution of $CuSO_4·5H_2O$ (94.0 mg, 0.380 mmol) and sodium ascorbate (76.0 mg, 0.380 mmol) was added. The reaction mixture was stirred for 10 h at 55 °C under a N_2 atmosphere. The reaction solvents were evaporated under vacuum. The residue was resolved by CH_2Cl_2

and purified by silica-gel column chromatography using CH_2Cl_2/CH_3OH (v/v, 30/1) as the eluent. GalAC4A-Ac (290 mg) was obtained with a yield of 65.0%.

^1H NMR (400 MHz, DMSO-d_6, δ)**:** 9.14 (t, J = 5.2 Hz, 4H, Triazole-N*H*), 7.98 (d, 8H, J = 8.0 Hz, Ar-H), 7.88 (s, 4H, Triazole-H), 7.81 (d, 8H, J = 12.0 Hz, Ar-H), 7.79 (s, 8H, calix-Ar-H), 5.22 (t, J = 5.2 Hz, 4H), 4.91-4.86 (m, 4H), 4.81 (d, 4H, J = 4.0 Hz), 4.74-4.69 (m, 4H), 4.53-4.49 (m, 16H), 4.19-4.14 (m, 4H), 4.11-3.91 (m, 20H), 3.70-3.59 (m, 8H), 2.01 (s, 12H, Ac-H), 1.97 (s, 12H, Ac-H), 1.91 (s, 12H, Ac-H), 1.88 (s, 12H, Ac-H) ppm.

^{13}C NMR (100 MHz, DMSO-d_6, δ): 170.48, 170.04, 170.01, 169.92, 165.95, 160.13, 154.32, 145.12, 135.27, 131.11, 128.88, 124.45, 122.20, 96.80, 69.05, 68.97, 66.21, 65.56, 62.14, 49.37, 35.19, 21.02, 20.96, 20.87 ppm.

MS (MALDI): $[M+Na]^+$: *m/z* calcd. for $C_{132}H_{144}N_{24}O_{48}Na^+$: 2855.96, found: 2856.05.

a

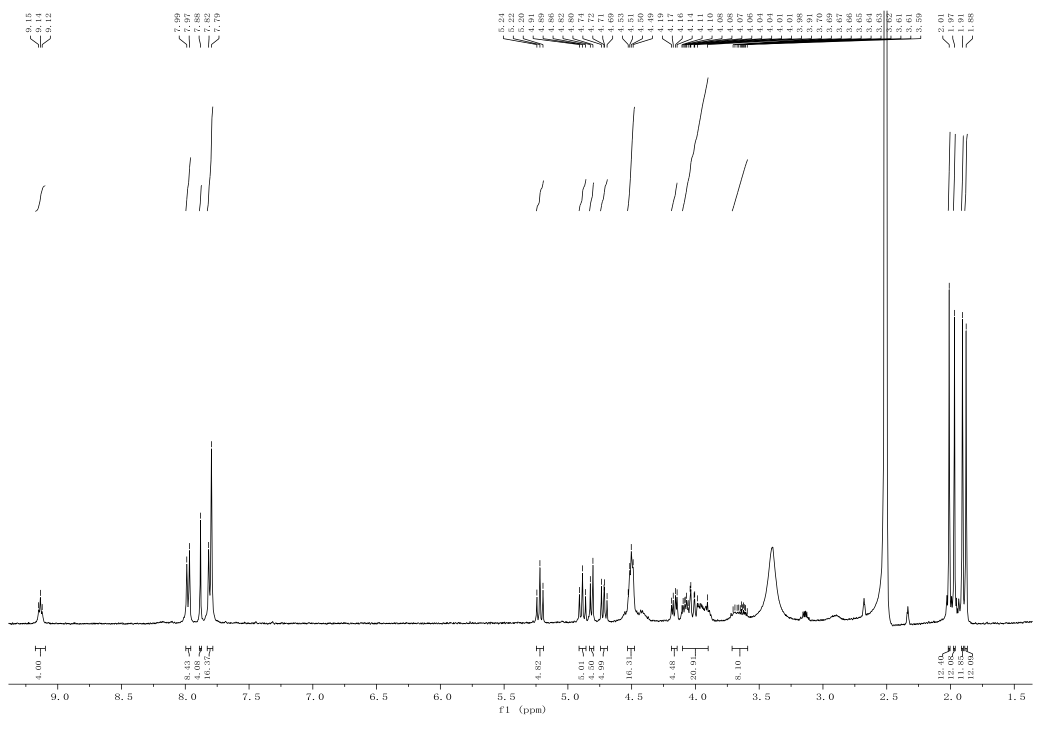

b

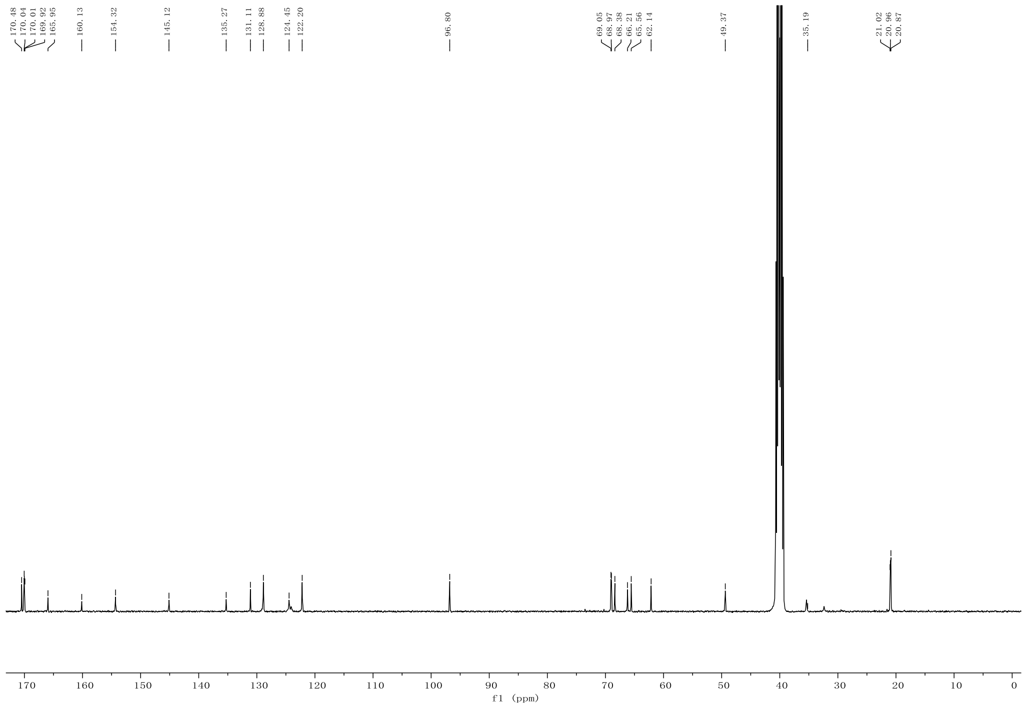

c

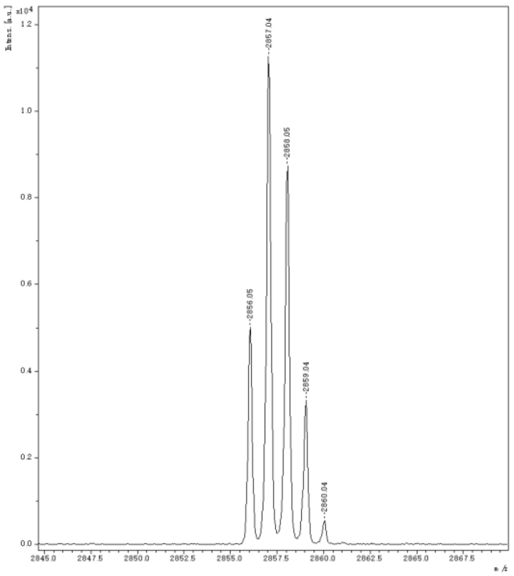

Supplementary Figure S7. Characterization of GalAC4A-Ac. (A) ^{1}H NMR spectrum of GalAC4A-Ac in DMSO-d_6, 400 MHz, 25 °C. (B) ^{13}C NMR spectrum of GalAC4A-Ac in DMSO-d_6, 100 MHz, 25 °C. (C) MS of GalAC4A-Ac.

GalAC4A: GalAC4A-Ac (200 mg, 0.0706 mM) and MeONa (92.0 mg, 1.68 mmol) were dissolved in anhydrous MeOH (20.0 mL). The reaction mixture was stirred at room temperature for 10 h. The reaction mixture was placed in a cellulose dialysis tube (cutoff 2000) and dialyzed against water for 2 days. The compound GalAC4A (137 mg)

was obtained through lyophilization with a yield of 90%.

^{1}H NMR (400 MHz, DMSO-d_6, δ)**:** 9.11 (t, J = 5.6 Hz, 4H, Triazole-N*H*), 8.04 (s, 4H, Triazole-H), 7.98 (d, 8H, J = 8.0 Hz, Ar-H), 7.81 (d, 8H, J = 8.0 Hz, Ar-H), 7.80 (s, 8H, calix-Ar-H), 6.64 (s, 24H, fumaric acid-C*H*=C*H*), 4.92-4.89 (m, 4H), 4.75-4.70 (m, 4H), 4.82-4.50 (m, 24H), 4.43-4.37 (m, 8H), 4.16 (d, 4H, J = 6.8 Hz), 4.09-4.03 (m, 8H), 3.90-3.84 (m, 8H) ppm. The purity was 100%.

^{13}C NMR (100 MHz, DMSO-d_6, δ): 166.03, 160.39, 154.31, 145.15, 135.32, 131.14, 128.89, 124.48, 124.21, 122.22, 104.01, 75.84, 73.76, 70.83, 68.61, 67.61, 60.93, 50.08, 35.39.

FTMS (MALDI): [M+Na]$^{+}$: *m/z* calcd. for $C_{100}H_{112}N_{24}O_{32}Na^{+}$: 2183.7900, found: 2183.7795.

a

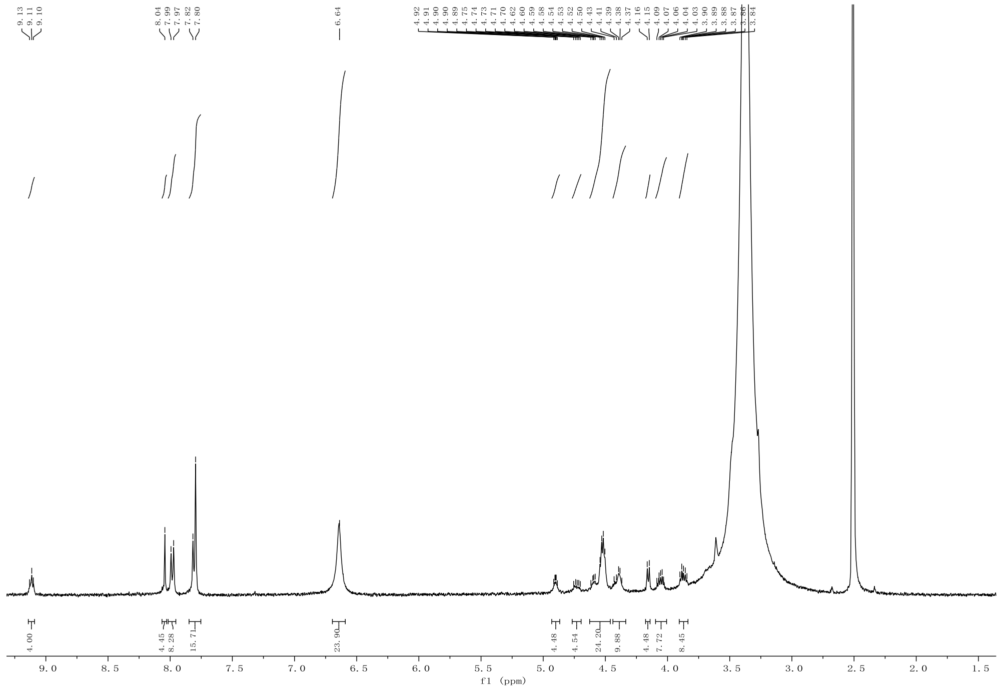

b

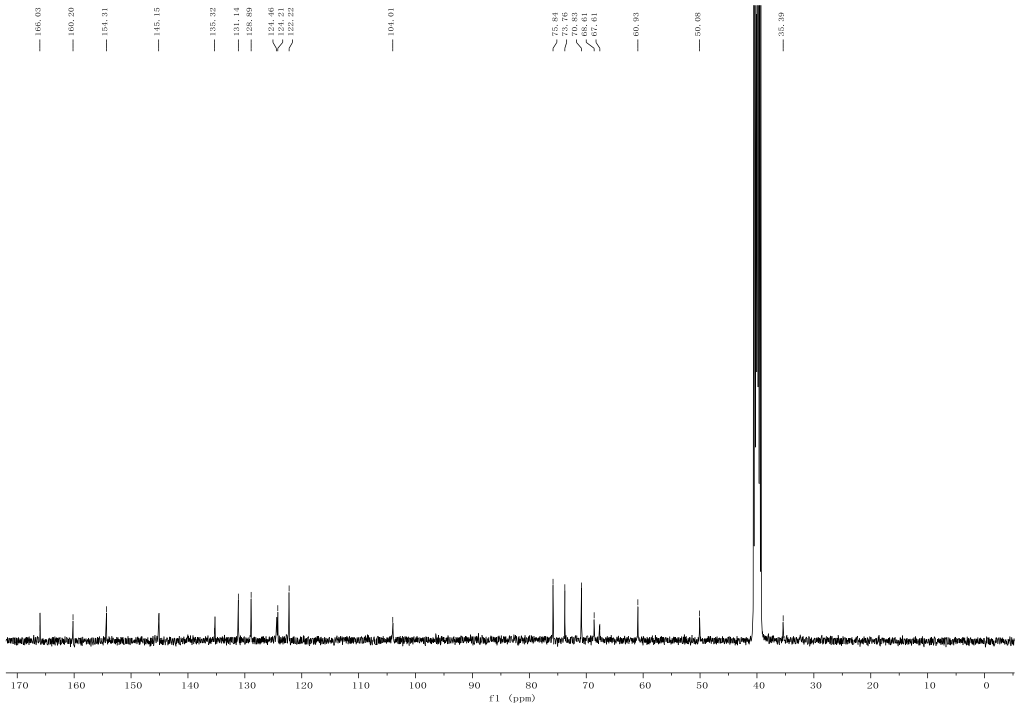

c

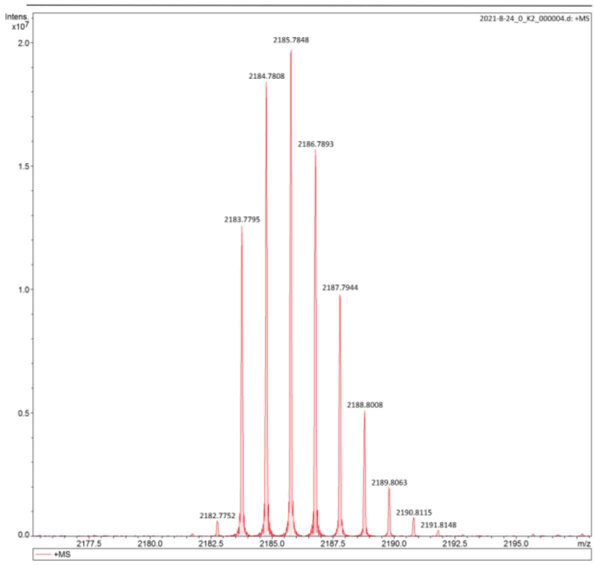

Supplementary Figure S8. Characterization of GalAC4A. (A) ^{1}H NMR spectrum of GalAC4A in DMSO-d_6, 400 MHz, 25 °C. (B) ^{13}C NMR spectrum of GalAC4A in DMSO-d_6, 100 MHz, 25 °C. (C) FTMS of GalAC4A.

3 Supplementary Figures

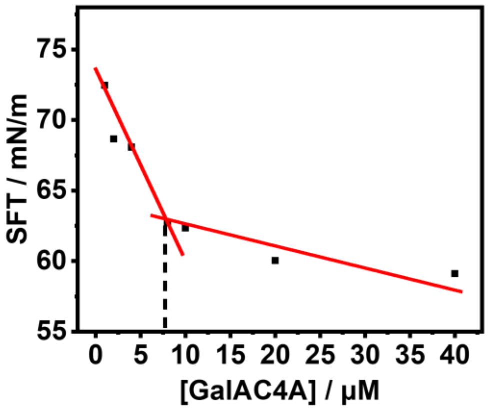

Supplementary Figure 9. The critical aggregation concentration (CAC) of GalAC4A.

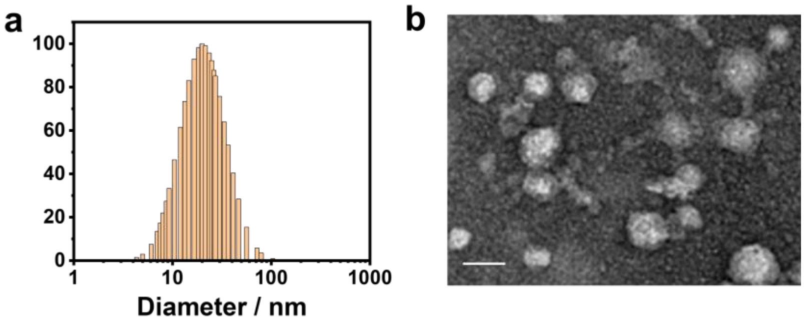

Supplementary Figure 10. The Size and morphology of GalAC4A. (a) DLS of GalAC4A (100 μM). (b) TEM image of GalAC4A. Scale bar was 20 nm.

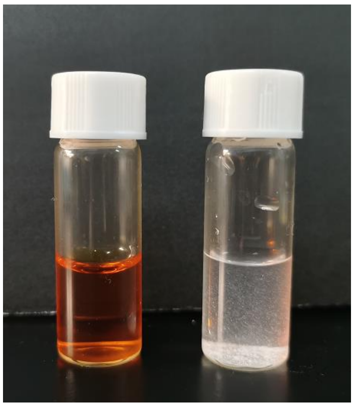

Supplementary Figure S11. The phase-solubility diagram picture of Cip with LacAC4A. The picture of Cip@LacAC4A (0.50/0.50 mM, left) solution and Cip (0.50 mM, right) solution.

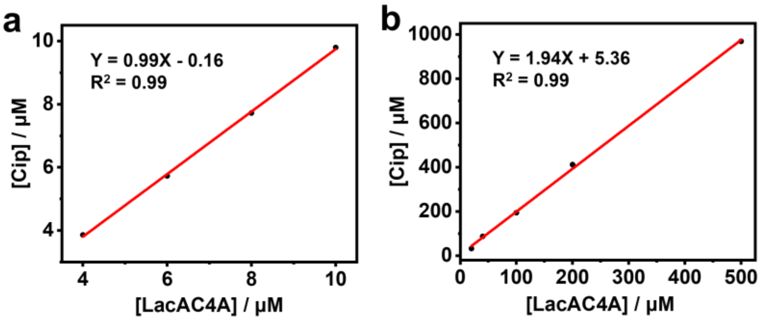

Supplementary Figure 12. The phase-solubility diagram of Cip with LacAC4A. The phase-solubility diagram of Cip with LacAC4A in PBS (10 mM, pH = 7.4) at 35 °C, the concentration of LacAC4A was 4 μM up to 10 μM (a) and the concentration of LacAC4A was 20 μM up to 0.50 mM (b). When the LacAC4A concentration was greater than 20 μM, the concentration of the solubilized Cip was twice of the calixarene concentration, which may be caused by LacAC4A self-assembly.

Supplementary Table S1. Parameters for phase-solubility diagrams of LacAC4A with Cip.

Host	Slope	CE[a]	[G]/[H][b]	K_a / M^{-1}[c]
LacAC4A[d]	0.99	99	99 : 100	$(6.70 \pm 1.09) \times 10^5$

[a] Complexation efficiency (CE) = [GH]/[H] = slope/(1–slope); [b] [G]/[H] = 1/(1+1/CE); [c] K_a = slope/(S_0(1–slope)); [d] The experiment was carried out in PBS solution (10 mM, pH = 7.4).

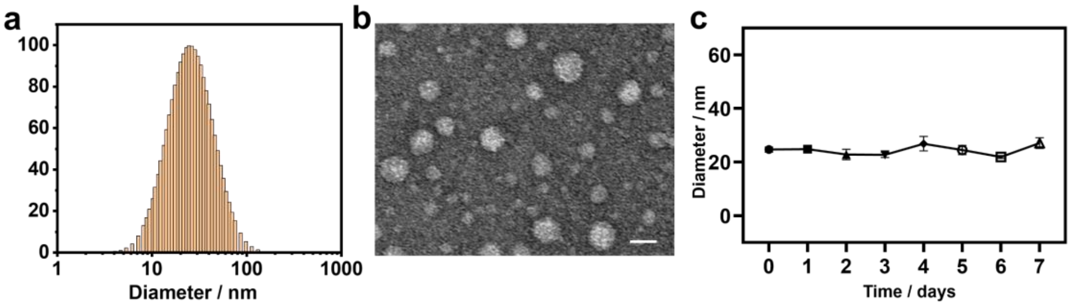

Supplementary Figure 13. Size, morphology and stability evaluation of Cip@LacAC4A. (a) DLS of Cip@LacAC4A (0.50/0.50 mM). (b) TEM image of Cip@LacAC4A (0.50/0.50 mM). Scale bar was 20 nm. (c) The stability of

Cip@LacAC4A (0.50/0.50 mM) in PBS (10 mM, pH = 7.4) at 4 °C by monitoring the size change for 7 days (n = 3). Data were presented as mean ± SD.

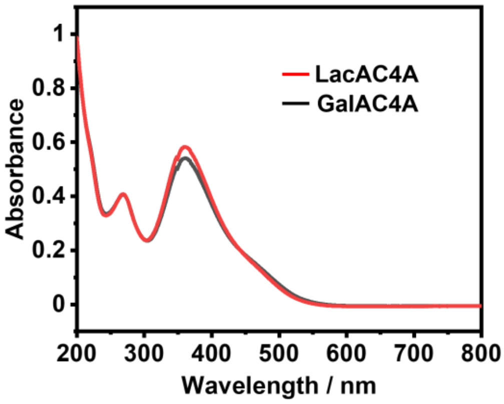

Supplementary Figure S14. UV-Vis absorption spectra of LacAC4A and GalAC4A. The UV−Vis absorption spectra of LacAC4A (10 μM) and GalAC4A (10 μM).

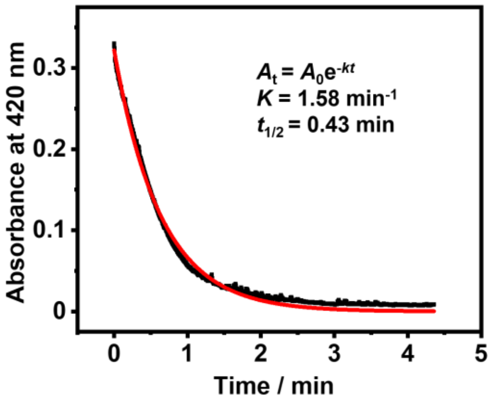

Supplementary Figure S15. The reduction kinetics fitting of LacAC4A. Absorbance (black) at 420 nm of LacAC4A (10 μM) as a function of time following the addition of SDT (10 mM) and the corresponding curve (red) was fitted according to a quasi-first order reaction decay model.

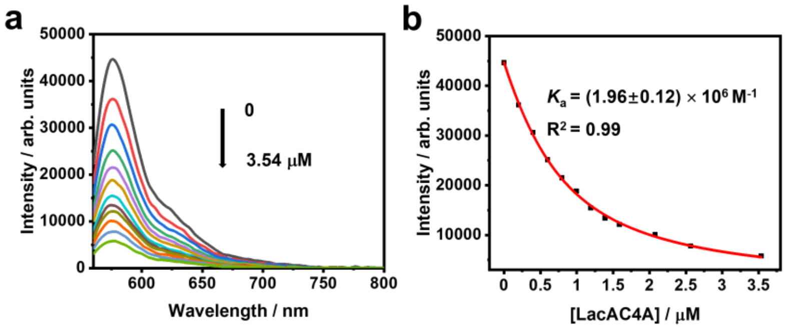

Supplementary Figure S16. Direct fluorescence titrations of RhB with LacAC4A. (a) Direct fluorescence titration of RhB (0.50 μM) with LacAC4A (up to 3.54 μM) in PBS (10 mM, pH = 7.4) at 25 °C, λ_{ex} = 554 nm. (b) The associated titration curve at λ_{em} = 575 nm was fitted according to a 1:1 binding stoichiometry.

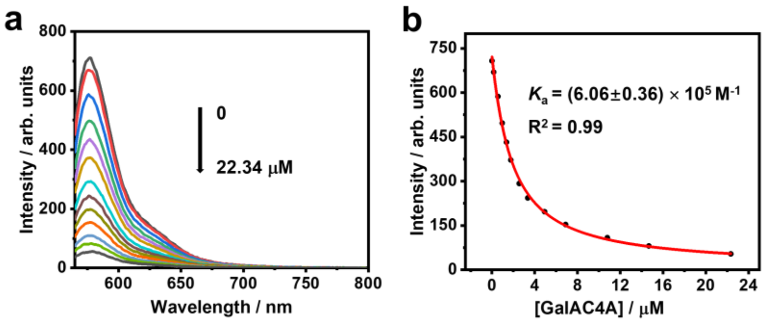

Supplementary Figure S17. Direct fluorescence titrations of RhB with GalAC4A. (a) Direct fluorescence titration of RhB (0.50 μM) with GalAC4A (up to 22.34 μM) in PBS (10 mM, pH = 7.4) at 25 °C, λ_{ex} = 554 nm. (b) The associated titration curve at λ_{em} = 575 nm was fitted according to a 1:1 binding stoichiometry.

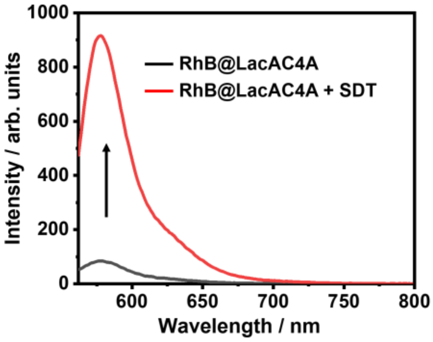

Supplementary Figure S18. The release of RhB@LacAC4A. Fluorescence spectra of RhB@LacAC4A (2.0/20 μM) before and after reduction by SDT (10 mM).

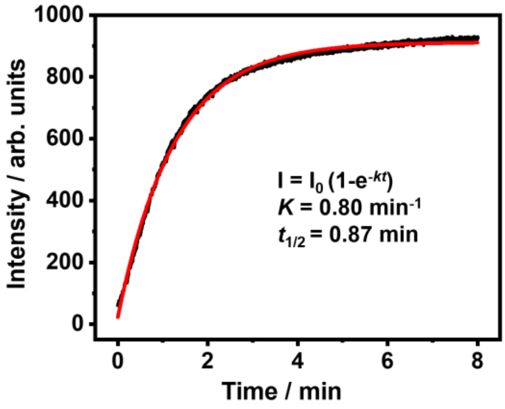

Supplementary Figure S19. The release kinetics of RhB@LacAC4A. Fluorescence intensity (black) at 575 nm of RhB@LacAC4A (2.0/20 μM) as a function of time following addition of SDT (10 mM), the corresponding curve (red) was fitted according to a quasi-first order reaction model.

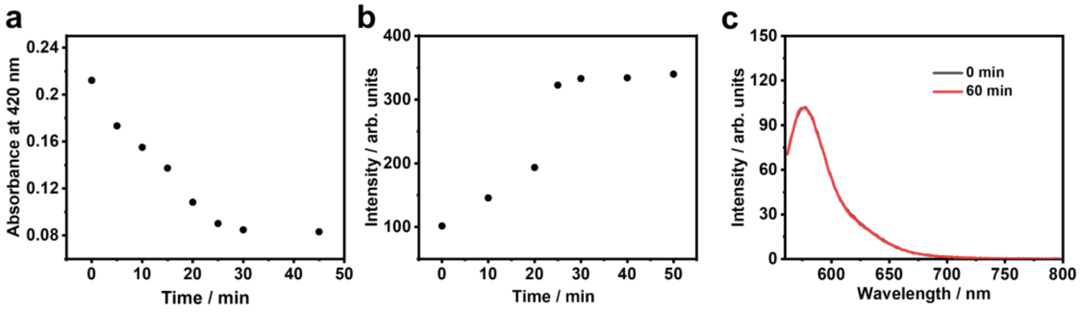

Supplementary Figure S20. The reduction of LacAC4A and the release of RhB@LacAC4A in the presence of rat liver microsomes. (a) Absorbance at 420 nm of LacAC4A (6.0 μM) as a function of time in the presence of rat liver microsomes (3.0 mg/mL) and NADPH (40 μM) under hypoxic conditions. (b) Fluorescence intensity at 575 nm of RhB@LacAC4A (1.0/5.0 μM) as a function of time in presence of rat liver microsomes (3.0 mg/mL) and NADPH (40 μM) under hypoxic conditions. (c) Fluorescence spectra of RhB@LacAC4A (1.0/5.0 μM) without rat liver microsomes and NADPH at 0 and 60 min. Experimental conditions: PBS (10 mM, pH = 7.4), 37 °C.

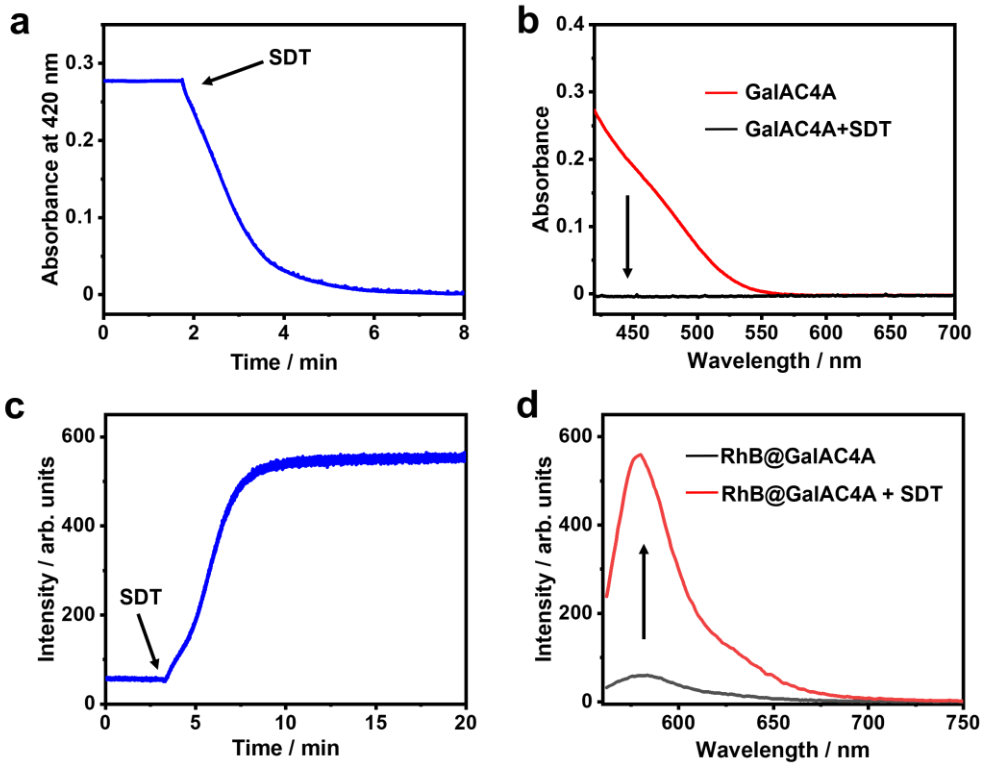

Supplementary Figure S21. The Reduction of GalAC4A and the release of RhB@GalAC4A. (a) Absorbance at 420 nm of GalAC4A (10 μM) as a function of time following addition of SDT (10 mM). (b) Absorbance spectra of GalAC4A (10 μM) before and after being reduced by SDT (10 mM). (c) Relative fluorescence intensity at 578 nm of RhB@GalAC4A (10/30 μM) at different time in the presence of SDT (10 mM). (d) Fluorescence spectra of RhB@GalAC4A (10/30 μM) before and after being reduced by SDT (10 mM). Experimental conditions: PBS (10 mM, pH = 7.4), 37 °C.

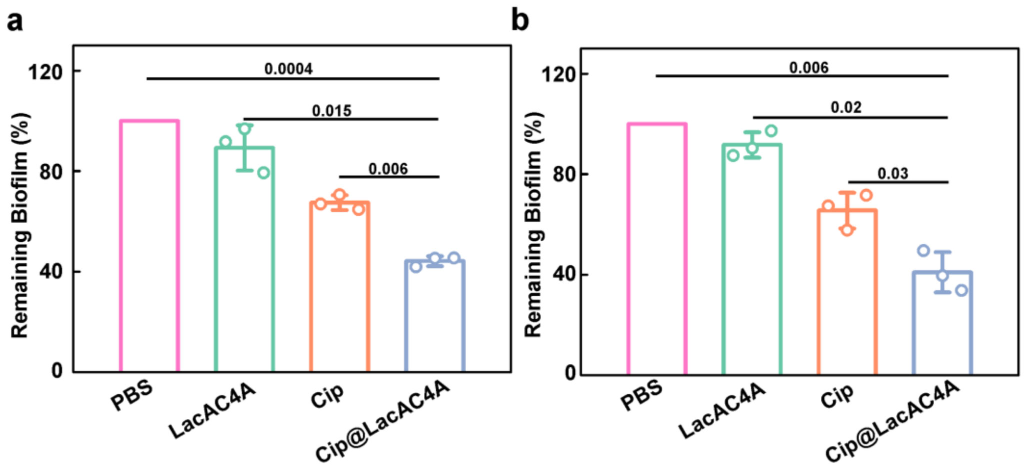

Supplementary Figure S22. Evaluation of the ability to disperse formatted biofilm.

(a) Dispersing percentage of MDR PA biofilm *in vitro*. (b) Dispersing percentage of PA 14 biofilm *in vitro*. Data were presented as mean ± SD, $n = 3$, * $p < 0.05$, ** $p < 0.01$, *** $p < 0.001$.

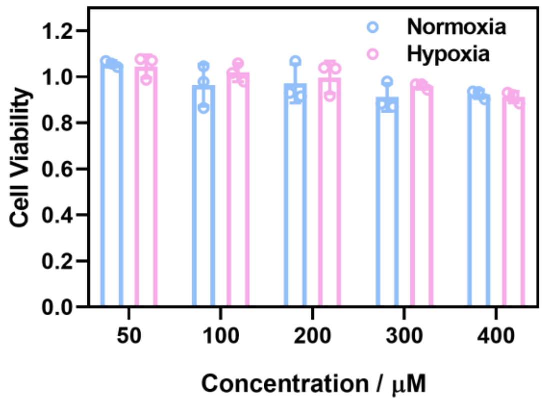

Supplementary Figure S23. Evaluation of the LacAC4A biocompatibility. The cytotoxicity of NIH 3T3 cells treated with LacAC4A at various concentrations under normoxic and hypoxic conditions. Data were presented as mean ± SD, $n = 3$.

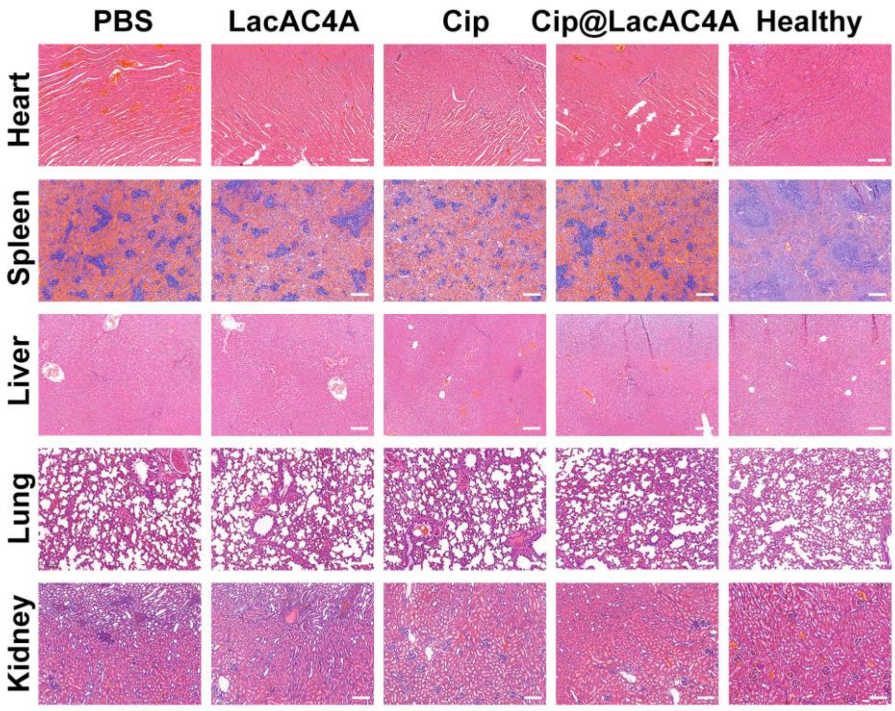

Supplementary Figure S24. Histological analysis of major organs after different treatments. Scale bar: 200 nm.

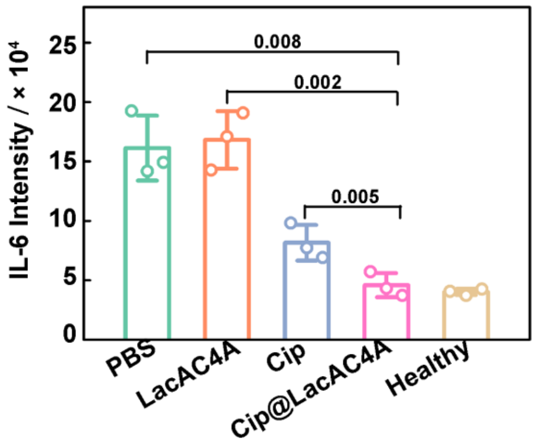

Supplementary Figure S25. Quantitative analysis of IL-6 in vivo. Quantitative analysis of IL-6 of wound tissues on 7 day. Data were presented as mean ± SD, $n = 3$, * $p < 0.05$, ** $p < 0.01$, *** $p < 0.001$.

4 Supplementary References

1. Guo, D.-S. Uzunova, V. D. Su, X. Liu, Y. & Nau, W. M. Operational calixarene-based fluorescent sensing systems for choline and acetylcholine and their application to enzymatic reactions. *Chem. Sci.* **2**, 1722−1734 (2011).

2. Ma, D. *et al.* Acyclic cucurbit[n]uril molecular containers enhance the solubility and bioactivity of poorly soluble pharmaceuticals. *Nat. Chem.* **4**, 503−510 (2012).

3. Loftsson, T. Hreinsdottir, D. & Masson, M. Evaluation of cyclodextrin solubilization of drugs. *Int. J. Pharm.* **302**, 18–28 (2005).

4. Loftsson, T. & Brewster, M. E. Pharmaceutical applications of cyclodextrins. 1. Drug solubilization and stabilization. *J. Pharm. Sci.* **85**, 1017−1025 (1996).

5. Zhang, Z. *et al.* Macrocyclic-amphiphile-based self-assembled nanoparticles for ratiometric delivery of therapeutic combinations to tumors. *Adv. Mater.* **33**, e2007719 (2021).

6. Hou, X. *et al*. Supramolecular radiosensitizer based on hypoxia-responsive macrocycle. *Adv. Sci.* **9**, e2104349 (2022).

7. Geng, W.-C. et al. A noncovalent fluorescence turn-on strategy for hypoxia imaging. *Angew. Chem. Int. Ed.* **58**, 2377−2381 (2019).
